# The Role of Adenosine A1 and A2a Receptors in Cerebral Blood Vessel Reactivity of Sprague Dawley Rats Exposed to Hyperbaric Oxygenation

**DOI:** 10.3390/molecules30142918

**Published:** 2025-07-10

**Authors:** Vedran Đambić, Zrinka Mihaljević, Ines Drenjančević, Ivana Jukić, Petar Šušnjara, Aleksandar Kibel

**Affiliations:** 1 Department of Physiology and Immunology, Faculty of Medicine Osijek, Josip Juraj Strossmayer University of Osijek, J. Huttlera 4, 31000 Osijek, Croatia; dambic.vedran@gmail.com (V.Đ.); zrinka.mihaljevic@mefos.hr (Z.M.); ines.drenjancevic@mefos.hr (I.D.); ivana.jukic@mefos.hr (I.J.); 2 Scientific Center of Excellence for Personalized Health Care, Josip Juraj Strossmayer University of Osijek, Trg Svetog Trojstva 3, 31000 Osijek, Croatia; psusnjara@kifos.hr; 3 Faculty of Kinesiology Osijek, Josip Juraj Strossmayer University of Osijek, 31000 Osijek, Croatia; 4 Scientific Unit for Medical Research, International Medical Center Priora, Kralja Tomislava 153, 31431 Cepin, Croatia; 5 Department of Clinical Medicine, Faculty of Dental Medicine and Health, Josip Juraj Strossmayer University of Osijek, Crkvena 21, 31000 Osijek, Croatia

**Keywords:** flow-induced dilation, hyperbaric oxygenation, adenosine receptors, cerebral blood vessels, hypoxia

## Abstract

Hyperbaric oxygenation (HBO_2_) can modify gene and protein expression, signaling pathways, and vascular function, leading to altered vasomotor responses. Adenosine receptors (ARs) may mediate these effects by modulating vasoactivity. This study investigated flow-induced dilation (FID) and hypoxia-induced dilation (HID) in the presence or absence of A1R/A2aR agonists (CCPA and CGS-21680, respectively) and antagonists (DPCPX and SCH-58261, respectively) in isolated middle cerebral arteries (MCAs) from Sprague Dawley rats of both sexes and the direct dose-dependent effects of A1R and A2aR agonists on the vascular reactivity of MCAs. Rats were exposed to either acute HBO_2_ (Ac-HBO_2_) or intermittent HBO_2_ over four days (In-HBO_2_). Ac-HBO_2_ impaired vascular responses to A1R and A2aR agonists and significantly decreased FID and HID. In both Ac-HBO_2_ and In-HBO_2_, A1R modulation did not significantly affect FID or HID. A2aR stimulation reduced FID in the In-HBO_2_ group, while A2aR antagonism had no significant effect on HID. However, the A2aR agonist’s presence enhanced HID in In-HBO_2_-exposed rats. Protein expression of A1R and A2aR decreased after Ac-HBO_2_, while gene expression increased following In-HBO_2_. These findings suggest that ARs play a role in HBO_2_-induced vasoreactivity, which possibly changes in MCA, potentially via the modulation of ARs gene and protein expression.

## 1. Introduction

Hyperbaric oxygenation (HBO_2_) affects the expression of genes and proteins, modulates signaling pathways at the molecular, cellular, and tissue levels, and affects vascular structure and function, which is of great importance in various physiopathological processes [1]. However, the impact of HBO_2_ on the regulation of the coordinated vascular system and microcirculation has not been fully clarified [2]. Importantly, the effects depend on the employed HBO_2_ protocols and the duration and frequency of exposure to HBO_2_. For example, acute HBO_2_ (Ac-HBO_2_) transiently impaired the dilation of blood vessels in response to hypoxia due to increased production of superoxide and an overall increase in oxidative stress, which was restored 24 h after treatment [3]. On the other hand, intermittent HBO_2_ (In-HBO_2_) improves vascular reactivity to stimuli such as angiotensin-(1-7) [ANG-(1-7)] and angiotensin II (ANG II) due to the activation of mechanisms related to CYP450 enzymes and the synthesis of epoxyeicosatrienoic acids (EETs) and the induction of antioxidant defense mechanisms [4,5]. Thus, it is hypothesized that acute exposure to HBO_2_ reduces the sensitivity and functional activity of A1R and A2aRs and thereby impairs vasoreactivity, while longer-term exposure to HBO_2_ leads to different adaptations in the expression level of A1Rs and A2aRs and changes in the enzymatic activity that regulates the conversion of adenosine and EETs, which could improve vasoreactivity.

The most important mediators of vasoreactivity in response to physiological stimuli such as flow-induced dilation (FID), hyperoxia, and hypoxia-induced dilation (HID) are various metabolites of arachidonic acid (i.e., prostaglandins, EETs, and 20-hydroxyeicosatetraenoic acid) and nitric oxide [6]. One of the main mediators responsible for vasodilation in HBO_2_-treated rats is 11,12-EET, the synthesis of which is increased by the activation of adenosine A2a receptors (A2aR) [7]. Adenosine, a metabolite of adenosine triphosphate (ATP), modulates the cellular function of endothelial cells by binding to specific P1 purinergic 7-transmembrane heterotrimeric G protein-coupled receptors (A1, A2a, A2b, and A3) [8]. Importantly, the concentration of adenosine in response to tissue oxygen levels is regulated by its synthesis through specific hydrolyzing enzymes named ectonucleoside triphosphate diphosphohydrolases CD39 and CD73 and its degradation by adenosine deaminase (ADA) or phosphorylation to adenosine monophosphate (AMP) by adenosine kinase [9]. Both receptors are represented in the vasculature and have a high affinity for adenosine, whereby A1R mediates vasoconstriction, while A2aR mediates vasodilatation with additional antiaggregating and anti-inflammatory effects [10,11]. HBO_2_, oxidative stress, and hypoxia may have an impact on the regulation of the adenosinergic pathway by regulating the activity of adenosine synthesis and degradation factors and may affect the expression of adenosine A2aRs [9]. Bruzzese et al. showed that hyperoxia decreases the expression of the A2aR gene in the brain, while the expression of the A2aR gene increases under hypoxic conditions [9,12]. Also, HBO_2_ itself changes the expression of the A2aR protein through the process of transcriptional/translational regulation [13]. Thus, adenosine A1Rs and A2aRs might play an important role in the mechanism of action of oxygen and carbon dioxide as reactive signaling molecules and vasoactive substances, and their gene and protein expression could possibly be affected by HBO_2_ and hypoxia.

In this study, we tested the hypothesis that Ac-HBO_2_ and In-HBO_2_ affect the FID and HID of MCAs of SD rats via A1R and A2aR. The aims of this study were to evaluate the effect of Ac-HBO_2_ and In-HBO_2_ on direct agonists’ dose–response stimulation of A1R and A2aR in isolated MCAs and to determine the gene and protein expression of adenosine A1R and A2aR. Second, we sought to determine the vascular reactivity of MCAs in response to FID and HID in the presence or absence of A1R or A2aR agonists and antagonists in control rats and rats treated with Ac-HBO_2_ and In-HBO_2_.

## 2. Results

### 2.1. General Data

Table 1 presents the general characteristics of rats used in the study, including body mass, the diameter of middle cerebral arteries (MCAs) under no-flow conditions (Δ0 mmHg), maximal diameter under Ca^2+^-free conditions, and active vascular tone. Data are shown for three experimental groups: CTRL (control group), Ac-HBO_2_ (acute hyperbaric oxygenation), and In-HBO_2_ (intermittent hyperbaric oxygenation). There were no statistically significant differences among the groups in any of the measured parameters.

### 2.2. Dose–Response Effects of A1R and A2aR Selective Agonists

**Figure 1:**
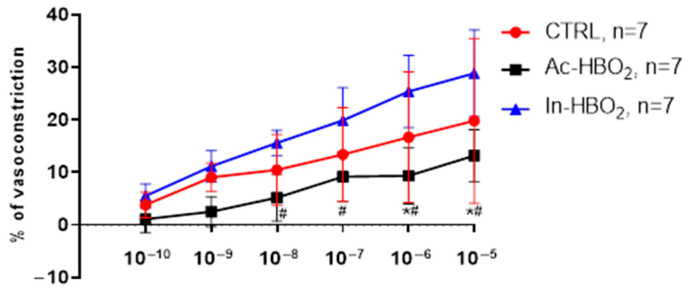
The response of the MCA of the CTRL, Ac-HBO_2,_ and In-HBO_2_ groups to A1R selective agonist CCPA, applied in stepwise concentrations 10^−10^–10^−5^ M. Data are presented as the mean ± SD. Significant differences were determined at ^#^ *p* < 0.05, Ac-HBO2 compared to In-HBO2; *^#^ *p* < 0.05, Ac-HBO_2_ compared to CTRL and In-HBO_2_, respectively; a two-way ANOVA test was performed (Table S1).

The vasoconstriction in response to A1R agonist CCPA was similar between the CTRL and In-HBO_2_ groups, while it was significantly reduced in the Ac-HBO_2_ group compared to the CTRL and In-HBO_2_ groups at concentrations of 10^−9^–10^−5^ M (Figure 1).

**Figure 2:**
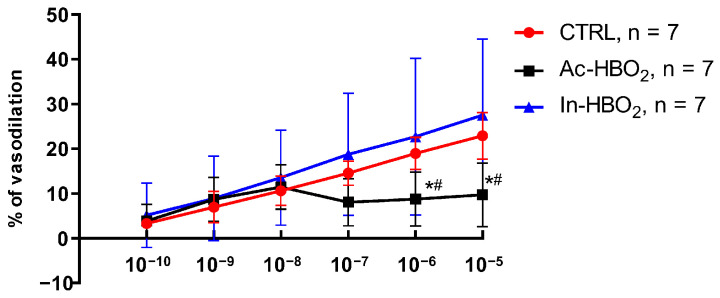
The response of the MCA of the CTRL rats exposed to Ac-HBO_2_ and In-HBO_2_ to the application of the A2aR agonist CGS-21680 in stepwise concentrations 10^−10^–10^−5^ M. Data are presented as mean ± SD. Significant differences were determined at *^#^ *p* < 0.05, Ac-HBO_2_ compared to CTRL and In-HBO_2_, respectively; a two-way ANOVA test was performed (Table S2).

The vasodilation in response to A2aR agonist CGS-21680 was significantly reduced in rats exposed to Ac-HBO_2_ compared to the CTRL and In-HBO_2_ groups at higher drug concentrations (10^−6^ to 10^−5^ M) (Figure 2).

### 2.3. Flow-Induced Dilation (FID) and the Use of Agonists and Antagonists of Adenosine A1 and A2a Receptors

**Figure 3:**
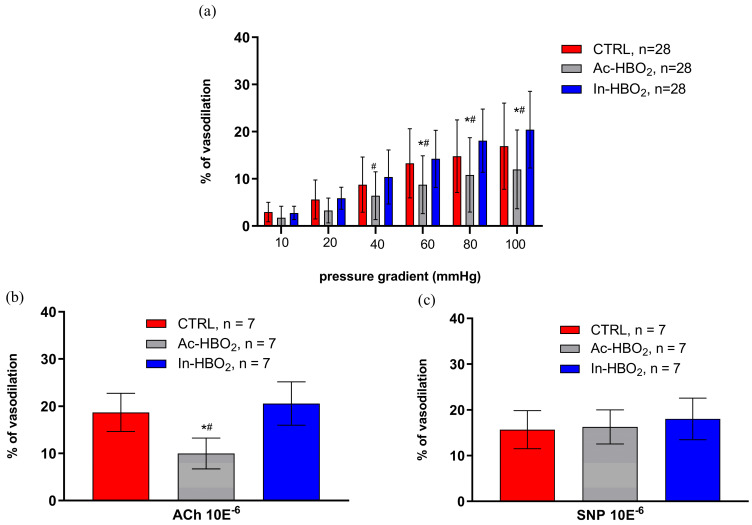
Flow-induced dilation (FID), acetylcholine-induced response (ACh), and sodium–nitroprusside (SNP)-induced response of middle cerebral arteries (MCAs). FID is presented as the percentage of dilatation (%) of the MCA in response to stepwise increases in the pressure gradient (∆10–∆100 mmHg) compared to baseline (no flow condition, ∆0 mmHg) (**a**). ACh (**b**) and SNP (**c**) were tested under no-flow conditions (at 80 mmHg). Data are presented as the mean ± SD. Significant differences were determined at ^#^ *p* < 0.05, Ac-HBO_2_ compared to In-HBO_2_; *^#^ *p* < 0.05, Ac-HBO_2_ compared to CTRL and In-HBO_2_, respectively. A two-way ANOVA ((**a**); Table S3a) or a one-way ANOVA test ((**b**,**c**); Table S3b,c) was performed, respectively.

**Figure 4:**
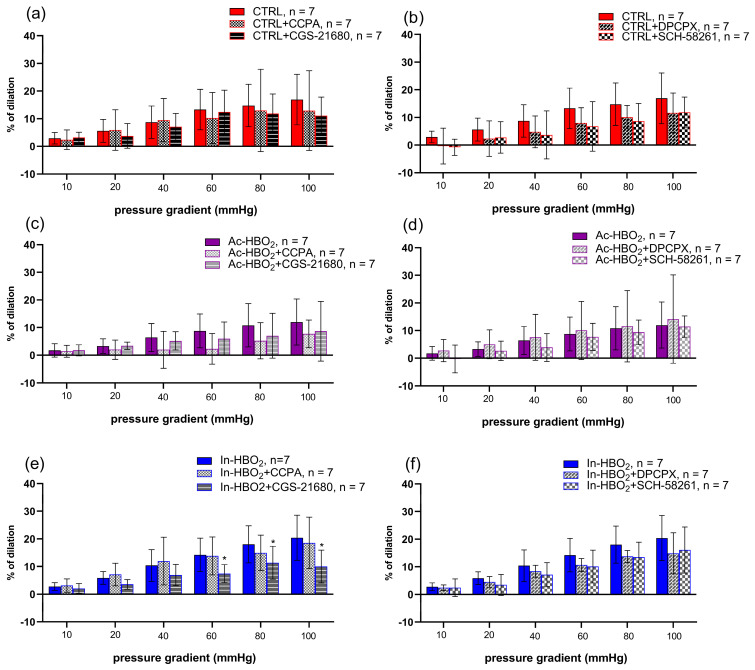
FID of the MCA in the presence of A1R and A2aR agonists (CCPA, 10^−6^ M and CGS-21680, 10^−6^ M, respectively) or antagonists (DPCPX, 10^−6^ M and SCH-58261, 10^−6^ M, respectively) in the CTRL (**a**,**b**), Ac-HBO_2_ (**c**,**d**), and In-HBO_2_ (**e**,**f**) groups of rats. The results are presented as the mean ± SD; * *p* < 0.05 compared to baseline. A two-way ANOVA test was performed (Table S4).

The FID was attenuated in the Ac-HBO_2_ group compared to the CTRL and In-HBO_2_ groups (Figure 3). The application of the agonist (CCPA) and antagonist (DPCPX) of A1Rs did not significantly affect FID in Ac-HBO_2_ and In-HBO_2_ (Figure 4). FID response during the presence of the A2aRs agonist (CGS-21680) in the In-HBO_2_ group was significantly reduced compared to baseline at pressure gradients of Δ60–Δ100 mmHg (Figure 4e). The application of the antagonist A2aRs (SCH-58261) did not significantly change FID in any of the experimental groups (Figure 4b,d,f).

### 2.4. The Role of AR1R and AR2aR in Hypoxia-Induced Vasodilation in the MCA of Rats Treated with HBO_2_

**Figure 5:**
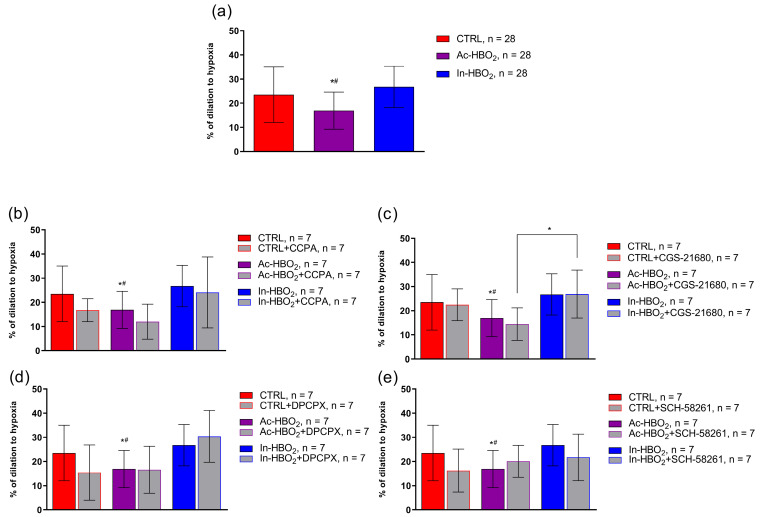
Hypoxia-induced dilation in the MCA of the CTRL, Ac-HBO_2,_ and In-HBO_2_ groups without (**a**) and with the A1R agonist—CCPA (**b**), A2aR agonist—CGS-21680 (**c**), A1R antagonist—DPCPX (**d**), and A2aR antagonist—SCH-58261 (**e**). The results are presented as the mean ± SD; *n*—number of rats; *^#^ *p* < 0.05, Ac-HBO_2_ compared to the CTRL and In-HBO_2_, respectively; * *p <* 0.05 Ac-HBO_2_+CGS-21680 compared to In-HBO_2_+CGS-21680; a one-way ANOVA test was performed (Table S5). Data are presented as the mean ± SD.

A significant decrease in hypoxia-induced dilation (HID) was observed in SD rats exposed to Ac-HBO_2_ without the presence of AR1 or AR2a agonists and antagonists (Figure 5a). There were no significant differences in HID after the administration of the adenosine AR1 agonist (CCPA) or antagonist (DPCPX) in the Ac-HBO_2_ or In-HBO_2_ groups compared to the CTRL (Figure 5b,d). There were no significant differences in HID in the presence of the adenosine AR2a agonist (CGS-21680) or antagonist (SCH-58261) in rats exposed to Ac-HBO_2_ compared to CTRL rats (Figure 5c,e). In the presence of an A2aR agonist (CGS-21680), HID was significantly more pronounced in rats exposed to In-HBO_2_ compared to Ac-HBO_2_ (Figure 5c). No significant difference in HID was observed in the presence of the AR2a antagonist (SCH-58261) in In-HBO_2_ compared to the other groups (Figure 5e).

### 2.5. Gene Expression of Adenosine A1 and A2a Receptors

**Figure 6:**
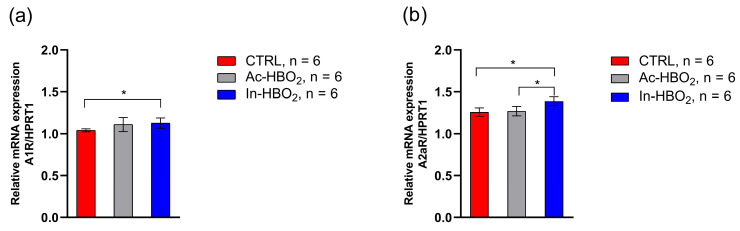
The relative gene expression of A1R (**a**) and A2aR (**b**) genes in cerebral blood vessels from the surface of the brain of the CTRL, Ac-HBO_2_, and In-HBO_2_ groups of rats determined using the RTqPCR method. Results are presented as the mean relative mRNA expressions normalized to the expression of the HPRT1 housekeeping gene. Significant differences were assessed as * *p* < 0.05. A one-way ANOVA test was performed (Table S6).

A1R gene expression was significantly increased in the In-HBO_2_ group compared to the CTRL group. The A1R gene expression between rats exposed to Ac-HBO_2_ and In-HBO_2_ was similar (Figure 6). In-HBO_2_ led to a significant increase in A2aR gene expression compared to the CTRL and Ac-HBO_2_. There was no difference in the gene expression of the A2aR gene between the CTRL and Ac-HBO_2_ groups.

### 2.6. Protein Expression of Adenosine A1 and A2a Receptors

**Figure 7:**
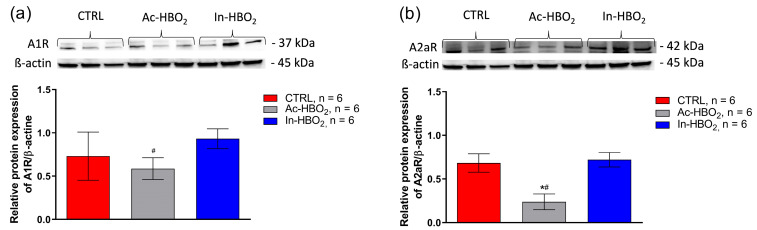
Relative protein expression and representative blots of A1R (**a**) and A2aR (**b**) in the surface cerebral blood vessels of the CTRL, Ac-HBO_2_, and In-HBO_2_ groups of rats determined using the Western blot method. Images were taken on a Bio-Rad ChemiDoc imager and analyzed using the ImageJ program. The results are presented as the mean relative protein expression normalized to the expression of β-actin. Significant differences were assessed as ^#^ *p* < 0.05, Ac-HBO_2_ compared to In-HBO_2_; *^#^ *p* < 0.05, Ac-HBO_2_ compared to CTRL and In-HBO_2_, respectively. A one-way ANOVA test was performed (Table S7). Data are presented as the means ± SD.

A1R protein expression was significantly reduced in Ac-HBO_2_ rats compared to In-HBO2 rats. The Ac-HBO_2_ group had significantly reduced protein expression of A2aR compared to the CTRL and In-HBO_2_ groups (Figure 7). The In-HBO_2_ group had similar protein expression of both adenosine receptors to the CTRL group.

The results obtained indicate that hyperbaric oxygenation (HBO_2_) induces significant alterations in vascular reactivity, manifested as modifications in the dilation or constriction of blood vessels by a certain percentage, depending on the specific adenosine receptor agonist. These changes in vascular tone contribute to the regulation of regional blood flow in targeted tissues, potentially affecting their oxygenation and metabolic function. The observed biological variability in responses suggests complex and multifactorial regulation of adenosine signaling pathways and vascular function during HBO_2_ exposure.

## 3. Discussion

The main findings of this study are as follows: (a) Ac-HBO_2_ significantly reduced A1R-mediated vasoconstriction and A2aR-mediated vasodilation compared to the control and In-HBO_2_ conditions; (b) furthermore, Ac-HBO_2_ significantly reduced the vascular response to FID and hypoxia but without changing the vascular response to FID and HID with the application of A1R and A2aR agonists and antagonists; (c) In-HBO_2_ leads to a decrease in the vasodilatory response to adenosine A2aR stimulation during FID, while it is significantly increased during HID; and (d) Ac-HBO_2_ exposure significantly reduced adenosine A1R compared to In-HBO_2_ and reduced A2aR protein expression compared to the CTRL and In-HBO_2_, while In-HBO_2_ exposure significantly increased A1R and A2aR gene expression. Overall, the results of this study suggest that differential exposure to HBO_2_ alters the vascular reactivity of the MCA of SD rats, which is modified by adenosine receptor agonist administration and affects adenosine receptor gene and protein expression in cerebral arteries.

Flow-induced dilation (FID) and hypoxia-induced dilation (HID) are important regulators of blood flow and tissue perfusion, and vascular oxidative stress can significantly change the vascular response of the middle cerebral artery (MCA) to these stimuli [14,15]. Our data are consistent with previous observations that acute exposure to HBO_2_ impairs the vascular response to FID and HID [3]. On the other hand, intermittent exposure may be beneficial for vascular relaxation [3,4,16,17,18]. In this study, there was no significant difference in vascular reactivity between the control group and rats exposed to In-HBO_2_ in response to FID and HID, in contrast with Ac-HBO_2_, which may be explained by the elimination of the increased oxidative stress in In-HBO_2_ treatments.

The role of adenosine A1 receptors (A1Rs) in the regulation of blood vessel tone differs among blood vessels of different orders [19]. For example, Bryan et al. showed that A1Rs and adenosine A2ARs contribute equally to adenosine-induced dilation in the skeletal muscle of Sprague Dawley (SD) rats [20]. The present study is the first study to confirm vasoconstriction by A1R activation in the MCA of SD rats. The results of dose–response stimulation by the A1R agonist suggest that HBO_2_ significantly contributes to the modification of this vascular response. Namely, Ac-HBO_2_ significantly impairs the vasoconstrictor response to direct administration of even the small dose (10^−9^ M) of the A1R agonist. Furthermore, we were interested in how additional A1R stimulation modifies the vascular reactivity of FID and HID that is altered by exposure to different HBO_2_ protocols. Application of A1R agonists and antagonists in HID and FID did not result in a significant difference in vascular reactivity in the Ac-HBO_2_ and In-HBO_2_ groups compared to control (CTRL). This may be explained by the fact that adenosine released during hypoxia causes cerebral vasodilatation by acting on A2ARs, while A1Rs have a minimal functional role in hypoxic conditions [21]. Ngai et al. also showed that A1R inhibition did not affect adenosine-induced dilation [22], which poses a question and calls for further investigation, whether A1R antagonism plays a role in vascular tone at all.

So far, HBO_2_ is known to act on different components of the adenosinergic pathway that act in concert to modulate the extracellular concentration of adenosine in response to oxygen levels [23]. HBO_2_ (increased oxygen concentration depending on the dose) significantly reduces adenosinergic pathways by reducing the concentration of adenosine in plasma by reducing the activity of endopeptidase CD73 (synthesis of adenosine) and increasing the activity of adenosine deaminase (degradation of adenosine) [9]. On the other hand, hypoxia increases the intracellular concentration and the concentration of adenosine in the plasma [24,25,26]. In mouse aortic smooth muscle, A1R stimulation is associated with 20-hydroxyeicosatetraenoic acid (20-HETE) production and protein kinase C (PKC) activation, which inhibits the activity of big potassium (BK) channels, resulting in vasoconstriction [27]. Given that reactive oxygen species (ROS) are also generated by the activation of adenosine receptors [28] and that ROS play a role in the modulation of BK channel activity [29,30], we can speculate that Ac-HBO_2_ modulates the vascular response via BK channels to A1R stimulation, but further research is needed to test this hypothesis.

It is known that the vasodilation response of cerebral arteries is certainly mediated by A2aR, while at higher concentrations of adenosine (>1 μM), A2bR may also contribute [22]. A2aR stimulation by agonist in this study resulted in a significant reduction in vasodilation to direct administration of the agonist in higher doses (10^−6^ and 10^−5^ M) in Ac-HBO_2_. This could be related to the production of different vasoconstrictor metabolites induced by A2aR. For example, various vasoactive metabolites of arachidonic acid (prostaglandins, EETs, and 20-HETE) produced by different CYP450 enzymes modulate vascular reactivity involving the adenosinergic pathway, particularly the adenosine–epoxygenase pathway [6,7,31]. It is known that impaired vasorelaxation in Ac-HBO_2_ in response to FID and hypoxia is mediated by increased synthesis of 20-HETE [6]. 20-HETE likely causes endothelial dysfunction by reducing NO release and increasing superoxide production [23]. Furthermore, 20-HETE, nitric oxide (NO), calcium-activated potassium channels, stretch-activated cation channels, and ROS play an important role in the modulation of vasomotor tone in response to changes in pressure and flow [32]. 20-HETE is a major mediator of flow-induced constriction in the MCA [32]. ROS, particularly those generated by CYP450 4A enzyme activation, play a role in flow-induced constriction in rat MCA by reducing NO bioavailability, leading to enhanced 20-HETE production [32]. Given that acute HBO_2_ has previously been shown to downregulate the A2aR-mediated adenosinergic pathway [9], the results of our study are in concordance with these findings, as in our study, there were no significant changes in vasoreactivity after A2aR activation in response to FID and hypoxia.

Intermittent exposure to HBO_2_ reduces the expression of endopeptidase CD26, which plays a key role in the breakdown of vasoconstrictor and antioxidant peptides, representing an adaptive response to high oxygen levels by increasing vascular tone and increasing antioxidant enzymes [9,33,34]. Suppression of the adenosinergic pathway mediated by A2aRs in acute hyperoxia inhibits the physiological mechanism of tissue protection mediated by A2aR [35,36]. In our study, we obtained a reduced vasodilatory response to stimulation with the A2aR agonist CGS-21680 in response to FID when the intravascular pressure difference was increased to 80 and 100 mmHg. It is known that an increase in intraluminal pressure and intraluminal flow (Δ flow) leads to flow-stimulated dilation, and at higher pressures, flow-stimulated constriction of the human and rat MCA as part of the autoregulation of cerebral blood flow [37,38]. This biphasic response of the MCA was also observed in our study in the presence of A2aR agonists (Figure 4). Also, several studies have confirmed that not only the large arteries (i.e., the MCA) but also the smaller MCA side branches (about 50 μm in diameter) constrict upon increasing pressure and flow [32,37,39]. Flow-induced constriction or dilatation of cerebral arteries in the autoregulation of cerebral flow requires complex regulatory mechanisms that are the interaction of vascular wall stretching and metabolic (e.g., adenosine) and chemical (e.g., changes in pCO_2_, pH, and pO_2_) factors [32].

The results of this study suggest that In-HBO_2_ improves the vascular response to hypoxia (compared to rats exposed to acute HBO_2_) with the activation of adenosine A2aRs, contrary to effects on FID. An increased vasodilatory response to hypoxia in rats exposed to intermittent HBO_2_ has previously been demonstrated to be connected to the activation of COX, with a consequent synthesis primarily of prostaglandin I2 (PGI_2_) and an activation of CYP450 epoxygenases and the formation of EET [6]. The increased ratio of vasodilators (primarily EET and PGI2) compared to vasoconstrictors (primarily 20 HETE) during intermittent exposure to HBO_2_ may represent the underlying mechanism of enhanced vasorelaxation [6]. In isolated arcuate arteries of SD rats, dilation at a pressure of 80 mmHg was mediated by A2aR activation and increased EET release [7,31]. EETs act as second messengers that, via activation of the Gsα protein, result in the opening of Ca^2+^-activated K^+^ channels in preglomerular microvessels [7]. One of the cerebral vasodilatation mechanisms is proposed through the stimulation of adenosine receptor subtypes A2A and A2B, whose activation by adenosine induces the formation of O^2−^ from NADPH oxidase and mitochondrial sources and the formation of its dismutation product H_2_O_2_, as demonstrated in isolated cerebral arterial muscle cells [40]. The generation of oxidants can strengthen the functional effects of these adenosine receptor subtypes by integrating with other released factors, such as NO, PGI_2_, and EETs or EDHF [41]. We assume that during hypoxic conditions, there is increased generation of the mentioned vasodilating factors and a significantly better vascular response to HID by activating the A2aR. This is in accordance with a study in which, in isolated feline MCAs, increases in flow at higher pressures led to a decrease in diameter (autoregulation), but the arteries dilated significantly under hypoxic conditions. Altogether, this implies that metabolic signals can override flow-induced constriction. Thus, hypoxia is one of the most powerful chemical inducers of gene expression, metabolic changes, and regenerative processes [42]. Acute exposure to HBO_2_ induces several hypoxic cellular mechanisms because fluctuations in the concentration of free oxygen, rather than the absolute level of oxygen, might be interpreted at the cellular level as a lack of oxygen, which is called the hyperoxic–hypoxic paradox (HHP) or intermittent pseudohypoxia [3,42]. It is HHP that has the most dominant effect on the expression of hypoxia-inducible factor 1 alpha (HIF-1α) [43]. HBO_2_ induces the expression of different types of HIF, and the dose–response curve is related to the applied pressure, time, and number of HBO_2_ exposures [42]. HIF-1α affects the expression of endothelial nitric oxide synthase (eNOS), inducible nitric oxide synthase (iNOS), heme oxygenase-1 (HO-1), cyclooxygenase-2 (COX-2), and the production of NO and prostaglandins under conditions of HHP, leading to an enhanced vasodilation response [42]. The increased vasodilatory response to hypoxia is the activation of cyclooxygenase (COX) with consequent synthesis of primarily PGI2 and activation of CYP450 epoxygenases and EETs [6] and increased expression of HIF1α [42]. Activation of A2a receptors may indirectly increase EET synthesis by activating cAMP and increasing the synthesis of CYP450 enzymes or their stability by inhibiting soluble epoxide hydrolase (sEH) enzymes that degrade EETs [8,44], which could contribute to a better vascular response, but perhaps not enough to override autoregulatory mechanisms (which are probably not mediated by A2aRs), except in the presence of a strong inducer of vasodilation such as hypoxia.

In this study, Ac-HBO_2_ led to decreased expression of A1R (compared to In-HBO_2_) and A2aR (compared to In-HBO_2_ and CTRL) at the protein level. The In-HBO_2_ group had similar protein expression of both adenosine receptors as the CTRL group. In-HBO_2_ significantly increased A1R (compared to CTRL) and A2aR (compared to CTRL and Ac-HBO_2_) gene expression. There was no difference in A2aR gene expression between the control and acute groups. In the present study, Ac-HBO_2_ led to decreased expression of A1R and A2aR at the protein level, while In-HBO_2_ increased gene expression of A2aR and maintained the gene and protein expression of A1R similar to the control. As acute exposure to HBO_2_ leads to increased synthesis of ROS, and it is known that ROS participate in the activation of transcription factors and the modulation of post-translational responses [45], we can assume that the potential changes in the protein expression of A1Rs and A2aRs are for the aforementioned reason. Our results—the reduction in A2aR protein expression after acute exposure to HBO_2_—are in accordance with the study conducted by Bruzzese et al., which confirmed that HBO_2_ itself significantly modulates the process of gene transcription and/or translation [9]. In-HBO_2_ increased A1R and A2aR gene expression, most likely representing an adaptive mechanism against the harmful effects of ROS and improvement of cerebral perfusion, given that both receptors are known to have neuroprotective roles [36].

**Figure 8:**
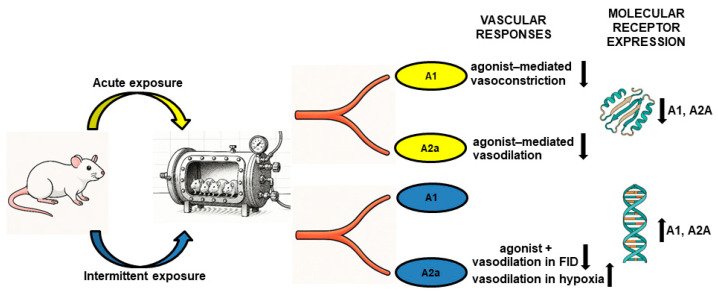
Summary figure. Rats were exposed to acute HBO_2_ (Ac-HBO_2_) or intermittent HBO_2_ over four days (In-HBO_2_). The main findings include impaired vascular responses to adenosine A1 receptor (A1R) and adenosine A2a receptor (2aR) agonists after Ac-HBO_2_. In both Ac-HBO_2_ and In-HBO_2_ groups, A1R modulation did not significantly affect flow-induced dilation (FID) and hypoxia-induced dilation (HID). A2aR stimulation reduced FID in the In-HBO_2_ group, while A2aR antagonism did not significantly affect HID. However, the presence of the A2aR agonist enhanced HID in In-HBO_2_-exposed rats. Protein expression of A1R and A2aR decreased after Ac-HBO_2_, while gene expression increased following In-HBO_2_. These findings suggest that ARs play a role in HBO_2_-induced vasoreactivity, which possibly changes in middle cerebral arteries (MCAs), potentially via modulation of AR gene and protein expression. Upward arrows indicate an increase in vascular response or in the protein and gene expression of adenosine A1 and A2a receptors, respectively. Downward arrows indicate a decrease in vascular response or in the protein and gene expression of adenosine A1 and A2a receptors, respectively.

With the present study, we have confirmed that under the conditions of acute exposure to HBO_2_, the administration of A1R and A2aR agonists reduced the vascular response without the presence of stimuli such as FID and HID, and the protein expression of both receptors was reduced. Furthermore, the administration of A2aR agonists with In-HBO_2_ reduces the vasodilatory response of vessels to FID (only at a higher gradient), while on the contrary, they increase HID at the same higher gradients, in conditions of increased expression of A1R and A2aR genes. Our results provide evidence in support of the hypothesis that additional stimulation or inhibition of adenosine A1R and A2aR modifies vascular reactivity that is altered under different HBO_2_ protocols. The various levels of tissue oxidative stress, antioxidant defense capacity, and antioxidative preconditioning in different exposure protocols of HBO_2_ might have different effects on the adenosinergic pathway. Changes in the ratio of vasoconstrictor and vasodilator metabolites at the cellular and molecular levels, including the influence of the interaction between EET and 20-HETE, which ultimately modulates vascular function, remain to be further investigated. The role of adenosine receptors in vascular reactivity has translational implications because a better understanding of the interaction between HBO_2_ and adenosine receptors creates a basis for more optimized decisions when using hyperbaric oxygenation in a therapeutic and clinical context (Figure 8).

## 4. Materials and Methods

### 4.1. Ethical Approval

All experimental procedures conformed with the European Guidelines for the Care and Use of Laboratory Animals (Directive 86/609) and were approved by the local and national Ethics Committee (Faculty of Medicine, University of Osijek; no. #2158-61-07-21-188, 15 November 2021; National Ethical Committee for the Protection of Animals Used for Scientific Purposes EPP348/2021, 10 December 2021; and Ministry of Agriculture, Croatia: no. 525-09/559-22-4, 1 April 2022). Animals were bred in the animal facility at the Faculty of Medicine Osijek, Josip Juraj Strossmayer University of Osijek, Osijek, Croatia, which is registered and certified as a user/breeder of mice and rats for educational and scientific purposes (No. HR-POK-005). The rats were housed in pairs in shoebox-style cages (IVC self-ventilating cages Tecniplast GR900) under standardized conditions, considering a temperature of 21–23 °C, and a humidity- and light-controlled room with free access to tap water and fed ad libitum with a commercially prepared pellet diet (Mucedola, Milan, Italy) and were maintained on a 12:12 h light–dark cycle.

### 4.2. Experimental Groups and Protocols for Exposure to HBO_2_

Healthy SD rats of both sexes (N = 120) at the age of 8–10 weeks were randomly divided into three groups. Untreated animals (controls, CTRL) and animals exposed to HBO_2_ in a hyperbaric chamber for experimental animals acutely (Ac-HBO_2_—one treatment of exposure to 100% oxygen at a pressure of 2 bars for 2 h, with additional 15 min for compression and decompression; the animals were sacrificed immediately after the end of decompression) and intermittently (In-HBO_2_—HBO_2_ exposure once a day for 4 consecutive days, sacrificed on the 5th day). To implement HBO_2_, a hyperbaric chamber with a volume of 110 L for laboratory animals Đuro Đaković Aparati d. d. was used, located in the Laboratory for Physiology of Circulation of the Faculty of Medicine in Osijek. The aforementioned protocol for exposure to HBO_2_ was standardized and was repeatedly used in our previous research [3,4,5,6,16,46,47].

### 4.3. Preparation of Isolated MCAs

The experimental protocol began with the weighing of SD rats and anesthetizing the animals with a combination of ketamine 75 mg/kg (Ketanest S 25 mg/mL, ampoules 2 mL, Pfizer Pharma GmbH, Berlin, Germany) and midazolam 2.5 mg/kg (Midazolam Torrex 5 mg/mL, 3 mL, Torrex Chiesi Pharma, Parma, Italy) administered intraperitoneally, followed by decapitation. Immediately after the sacrifice of each rat, the brain was promptly surgically isolated, and the MCA was prepared and placed in a pressure myograph chamber (Pressure Myograph System model 110P MyoView Version 1.2.0. company DMT-Danish Myo Technology, Hinnerup, Denmark). The MCA was mounted on two glass micropipettes (outer diameter ~100–200 µm) placed in a chamber filled with warm (37 °C) physiological saline (PSS, pH = 7.4 ± 0.05; composition (in mM/L): 119 NaCl, 4.7 KCl, 1.17 MgSO_4_, 1.6 CaCl_2_, 1.18 NaH_2_PO_4_, 24 NaHCO_3_, 0.026 EDTA, and 5.5 glucose). The system was continuously oxygenated with a gas mixture of 21% O_2_, 5% CO_2,_ and the balance of N_2_ or with a gas mixture of 0% O_2_, 5% CO_2,_ and the balance of N_2_ (HID), depending on the research protocol. After placement in the chamber, the artery was incubated for 60 min with intravascular pressure maintained at 80 mmHg to estimate the baseline (basal) diameter. The vessel was recorded at all times with an infrared camera with a clear image on the monitor in order to precisely measure the change in diameter of the vessel between the inner edges of the endothelium. In both protocols, rats were sacrificed randomly from different groups, and adenosine A1R and A2aR agonists and antagonists were randomly used (N = 7 per group).

### 4.4. Determination of the Dose–Response of Adenosine A1 and A2a Receptor Agonists

After a 60 min incubation of the vessel, the smallest dose of adenosine A1R (2-Chloro-N6-cyclopentyladenosine (CCPA), Abcam, Cambridge, UK, 10^−10^ M) or A2aR agonists ((3-[4-[2-[[6-amino-9-[(2R, 3R, 4S, 5S)-5-(ethylcarbamoyl)-3,4-dihydroxyoxolan-2-yl] purin-2-yl] amino] ethyl] phenyl] propanoic acid (CGS 21680), Abcam, Cambridge, UK, 10^−10^ M) was added to the chamber underflow with intravascular pressure maintained at 80 mmHg, and after 15 min of agonist incubation, the diameter of the vessel was measured. The procedure was repeated until the highest determined drug concentration (10^−5^ M). Before adding the next dose of the agonist, the PSS in the chamber was replaced with fresh PSS. The dose–response of each agonist was determined in all three groups: CTRL, Ac-HBO_2_, and In-HBO_2_.

### 4.5. FID and Hypoxia-Induced Dilation (HID) in Isolated MCAs

After 60 min incubation, the blood vessel was exposed to flow, which was achieved by simultaneous changes in inflow and outflow pressure (pressure gradients Δ0, Δ10, Δ20, Δ40, Δ60, Δ80, and Δ100 mmHg). Each pressure gradient resulted in a change in the flow of the PSS solution through the placed blood vessel, and the diameter of the vessel was recorded at each of the specified gradients. To test HID, after stabilization, the vessel was exposed to hypoxia (the gas mixture was switched from 21% O_2_ to 0% O_2_) for 20 min, and the diameter of the vessel was measured at the pressure gradient of Δ0 (80 mmHg inflow and outflow pressure). FID and HID were tested prior (to asses baseline values) and after 30 min incubation of A1R and A2aR agonists (CCPA, Abcam, 10^−6^ M and CGS-21680, Abcam, 10^−6^ M, respectively) or antagonists (8-Cyclopentyl-1,3-dipropylxanthine (DPCPX), Abcam, Cambridge, UK, 10^−6^ M and 5-Amino-7-(2-phenylethyl)-2-(2-furyl)-pyrazolo (4,3-e)-1,2,4-triazolo (1,5-c) pyrimidine (SCH-58261), Abcam, Cambridge, UK, 10^−6^ M, respectively). The compounds CCPA, CGS-21680, DPCPX, and SCH-58261 were selected for this study due to their high receptor selectivity, particularly in the context of endothelial function, well-characterized pharmacodynamic profiles, and widespread standard use as reference ligands for adenosine A1 and A2a receptors, which guarantees reliable and comparable results [48,49,50,51].

### 4.6. Acetylcholine-Induced Dilation, Endothelium-Independent Dilation, and Maximum Diameter Measurements

Acetylcholine (ACh)-induced dilation was tested at the beginning of each experiment, and the direct NO donor sodium nitroprusside (SNP) was used to determine endothelium-independent dilation at the end of the experiments. Following the SNP protocol, the PSS solution in the system was replaced with a Ca^2+^-free PSS (pH = 7.4 ± 0.05; composition (in mM/L): 119 NaCl, 4.7 KCl, 1.17 MgSO_4_, 1.18 NaH_2_PO_4_, 24 NaHCO_3_, 0.026 EDTA and 5.5 glucose) solution to measure the blood vessel’s maximum diameter. Active tone (in %) was calculated as follows: [(D_max_ − D_bas_)/D_max_] × 100, where D_max_ and D_bas_ are the maximum and baseline diameters (Δ0 mmHg, without flow) of the vessel, respectively.

### 4.7. mRNA Expression Experiments

The gene expression of the adenosine receptors was determined using the RT-PCR method (Bio Rad CFX96, Hercules, CA, USA) from the collected superficial brain blood vessels (BBVs). After isolation, blood vessel samples were frozen in liquid nitrogen and stored at −80 °C until further processing. Total RNA was extracted using TRI Reagent (Molecular Research Center, INC), according to the manufacturer’s instructions. RNA concentration and purity were checked using a NanoDrop (Implen, Munich, Germany). Total RNA was additionally purified from DNA using a Deoxyribonuclease kit (Sigma-Aldrich, Darmstadt, Germany) following the kit manufacturer’s instructions. Reverse transcription was performed using the High-Capacity cDNA Reverse Transcription kit (Applied Biosystems, Carlsbad, CA, USA), according to the manufacturer’s instructions on a C1000 Touch thermal cycler (BioRad CFX96, Hercules, CA, USA). Expression was determined by using uniquely designed (‘custom made’) primers on the Primer Express system (Applied Biosystems, A1 receptor forward 5′-TTCCAGGCTGCCTACATTGG-3′, reverse 5′-ATGGAGCTCTGGGTGAGGAT-3′; A2a receptor forward 5′-GCAGCGCTAGTTTCGAAGTC-3′, reverse 5′-CTCGAACAGACAGGTCACCC-3′) using ABsolute QPCR SYBR Green low ROX master mix-a (Thermo Scientific, Rockford, IL, USA). Gene expression was normalized to the HPRT “housekeeping gene” (forward 5′-GAAAGAACGTCTTGATTGTTGAAGATAT-3′, reverse 5′-GAGAGGTCCTTTTCACCAGCAA-3′).

### 4.8. Protein Levels of Adenosine A1 and A2a Receptors

Protein levels of adenosine A1Rs and A2aRs were determined using the Western blot method in BBVs. BBVs upon isolation were immediately frozen in liquid nitrogen and stored at −80 °C until homogenization. Vessels from two rats in the same treatment group were pooled into one sample to ensure sufficient protein yield for Western blot analysis. Samples were first pulverized in liquid nitrogen, and homogenates of tissue samples were prepared on ice with an ULTRA-TURARAX homogenizer using a homogenization buffer containing 10 mmol/L Tris base (Sigma-Aldrich, Darmstadt, Germany), 1 mmol/L EDTA (Sigma-Aldrich, Darmstadt, Germany), 0.4% SDS (Acros Organic, Geel, Belgium), and proteinase inhibitor cocktail (4 µL/100 µ, Sigma Aldrich, Darmstadt, Germany). Before determining the concentration of total proteins, the homogenates were centrifuged at 17,000 rpm, 30 min at 4 °C, and the total protein concentration was determined in the supernatants using the Bradford test (AppliChem, Darmstadt, Germany) according to the manufacturer’s instructions and stored at −80 °C until further use. Protein expression of adenosine A1Rs and A2aRs was determined using the Western blot method, which consists of electrophoresis (for the purpose of separating proteins on a gel according to size), protein transfer from the gel to a PVDF membrane, and detection of target proteins using specific antibodies. Before primary (4 °C overnight) and secondary (2 h at room temperature) antibody incubation, membranes were blocked for 2 h in 50 mL blocking solution (5% nonfat milk powder solution in TBST, room temperature). Protein levels were assessed using appropriate primaries (Rabbit polyclonal anti-rat adenosine A1R antibody, Abcam, ab82477; Rabbit polyclonal anti-rat adenosine A2aR antibody, Abcam, ab3461; β-actin-HRP, mouse MonoAb, Abcam, ab49900) and a secondary antibody (goat anti-rabbit HRP, Abcam, ab205718). Detection was performed with the chemiluminescence method using a Pierce ECL Western Blotting substrate (Thermo Scientific, Rockford, IL, USA) according to the manufacturer’s instructions, and the signal was recorded using a BioRad ChemiDoc MP Imaging System (BioRad, Hercules, CA, USA). The obtained images were processed and analyzed with ImageJ 1.52a software (National Institutes of Health, Bethesda, MD, USA) [52] according to the instructions of the software developer, and protein expression was determined as relative expression in relation to β-actin, which was also used as a loading control.

### 4.9. Statistical Analysis

FID results were analyzed with a two-way ANOVA test followed by Tukey’s post hoc test. For the results of HID, ACh, SNP, and gene and protein expression, the one-way ANOVA test was used, followed by the Holm–Sidak or Kruskal–Wallis test. For individual results, the Student’s *t* test was used to determine the mutual difference of normally distributed numerical variables between two independent groups, and in the case of deviations from the normal distribution, the Mann–Whitney U test was used. A test power of 0.8 with a *p*-value < 0.05 and a minimum expected difference of 0.25 indicated that at least 4 animals/group were required. SigmaPlot v.12 (Systat Software, Inc., Chicago, IL, USA) and GraphPad Prism, version 6.01 for Windows (GraphPad Software, Boston, MA, USA), were used for statistical analysis. The results are presented as the mean value + SD, and the level of significance was determined at *p* < 0.05. A tabular presentation of the statistical analysis is attached in the supplement as Supplementary File S1.

## 5. Conclusions

The results suggest that HBO_2_ modulates the vascular responses of the MCA to adenosine receptor agonists, implying an important role of A1R and A2aR in changes in vascular reactivity induced by HBO_2_. Furthermore, HBO_2_ may contribute to changes in MCA vasoreactivity in SD rats in response to FID and hypoxia, potentially by altering the gene and/or protein expression of adenosine receptors. Our results suggest and confirm the hypothesis of the influence of hyperbaric oxygen in different HBO_2_ exposure protocols on the adenosinergic pathway, which needs to be further investigated at the cellular and molecular level. Also, the level of tissue oxidative stress, antioxidant defense capacity, and antioxidative preconditioning in different protocols of exposure to HBO_2_ on the adenosinergic pathway and changes in the ratio of vasoconstrictor and vasodilator metabolites at the cellular and molecular levels remain to be investigated together with the mutual influence of EET and 20-HETE as two strong opposite players, which ultimately modulates vascular function. The role of adenosine receptors in vascular reactivity has translational implications because it creates a basis for optimal decisions when using hyperbaric oxygenation in a therapeutic and clinical context.

### Study Limitations

Despite the insights provided by this study, further research is needed to better understand the role of reactive oxygen species (ROS) in the balance between oxidative stress and antioxidant defense mechanisms, especially in the context of hyperbaric oxygenation. This interaction can significantly affect the signaling of adenosine receptors by altering their expression, affinity, and function. There is still insufficient data on tissue-specific differences in response to HBO_2_ and on the molecular mechanisms through which adenosine receptors mediate their effects. Therefore, additional studies are required to more precisely clarify the role of individual receptor subtypes, particularly under various physiological and pathological conditions of hyperoxic stress exposure, which could have important implications for optimizing the therapeutic use of hyperbaric oxygenation.

## Figures and Tables

**Table 1:** Body mass of rats, resting diameter, maximal diameter, and active tone of MCAs used in experiments.

	CTRL	Ac-HBO_2_	In-HBO_2_
Body mass [g]	372 ± 53	350 ± 44	349 ± 60
Diameter of MCAs at ∆0 mmHg [μm]	133 ± 23	123 ± 26	125 ± 23
Max. diameter of MCAs (Ca^2+^ free) [μm]	207 ± 19	190 ± 11	207 ± 14
Active tone (%)	43 ± 8	40 ± 6	42 ± 10

Data are presented as the mean ± standard deviation; *n* = 120 rats. CTRL, control group; Ac-HBO_2_, acute hyperbaric oxygenation; In-HBO_2,_ intermittent hyperbaric oxygenation; *p* > 0.05, one-way ANOVA test.

## Data Availability

The original contributions presented in this study are included in the article. Further inquiries can be directed to the corresponding author(s).

## Supplementary Materials

The following supporting information can be downloaded at https://www.mdpi.com/article/10.3390/molecules30142918/s1, Table S1: Statistical analysis of the response of the MCA of the CTRL, Ac-HBO_2_ and In- HBO_2_ groups to A1R selective agonist CCPA, applied in stepwise concentrations 10^-10^–10^−5^ M.; Table S2: Statistical analysis of the response of the MCA of the CTRL, rats exposed to Ac-HBO_2_ and In- HBO_2_, to the application of the A2aR agonist CGS-21680 in stepwise concentrations 10^-10^–10^−5^ M; Table S3: Statistical analysis of the flow-induced dilation (FID, Table S3a), acetylcholine-induced response (Ach, Table S3b), and sodium-nitroprusside (SNP, Table S3c)-induced response of middle cerebral arteries (MCA); Table S4: Statistical analysis of the FID of MCA in the presence of A1R and A2aR agonists (CCPA, 10^−6^ M and CGS-21680, 10^−6^ M, respectively) or antagonists (DPCPX, 10^−6^ M and SCH-58261, 10^−6^ M, respectively) in the CTRL (S4a and S4b), Ac-HBO_2_ (S4c and S4d) and In-HBO_2_ (S4e and S4f) groups of rats; Table S5: Statistical analysis of the hypoxia-induced dilation (FID) in the MCA of the CTRL, Ac-HBO_2_ and In-HBO_2_ groups without (S5a) and with A1R agonist—CCPA (S5b), A2aR agonist—CGS-21680 (S5c), A1R antagonist – DPCPX (S5d) and A2aR antagonist—SCH-58261 (S5e); Table S6: Statistical analysis of the relative gene expression of A1R (S6a) and A2aR (S6b) genes in cerebral blood vessels from the surface of the brain of CTRL, Ac-HBO_2_ and In-HBO_2_ groups of rats determined by RTqPCR method; Table S7: Statistical analysis of the relative protein expression of A1R (S7a) and A2aR (S7b) in surface cerebral blood vessels of CTRL, Ac-HBO_2_ and In-HBO_2_ groups of rats determined by Western blot method.

## Abbreviations

The following abbreviations are used in this manuscript:

ACh	acetylcholine
Ac-HBO_2_	acute hyperbaric oxygenation
A1R	adenosine A1 receptor
A2a	adenosine A2a receptor
ADA	adenosine deaminase
AMP	adenosine monophosphate
ARs	adenosine receptors
ATP	adenosine triphosphate
ANG-(1-7)	angiotensin-(1-7)
BK	big potassium channels
COX-2	cyclooxygenase-2
CTRL	control group
DMT	Danish Myo Technology
eNOS	endothelial nitric oxide synthase
EDHF	endothelium-derived hyperpolarizing factor
EET	epoxyeicosatrienoic acid
FID	flow-induced dilation
HO-1	heme oxygenase-1
H_2_O_2_	hydrogen peroxide
HBO_2_	hyperbaric oxygenation
HHP	hyperoxic-hypoxic paradox
HID	hypoxia-induced dilation
HIF-1α	hypoxia-inducible factor 1 alpha
iNOS	inducible nitric oxide synthase
In-HBO_2_	intermittent hyperbaric oxygenation
MCA	middle cerebral artery
PGI_2_	prostaglandin I 2
PKC	protein kinase C
ROS	reactive oxygen species
SNP	sodium nitroprusside
SD rats	Sprague Dawley rats

## References

[1] Drenjancevic I., Kibel A. (2014). Restoring vascular function with hyperbaric oxygen treatment: Recovery mechanisms. J. Vasc. Res..

[2] Drenjancević-Perić I., Gros M., Kibel A. (2009). Influence of hyperbaric oxygen on blood vessel reactivity: Concept of changes in conducted vasomotor response. Coll. Antropol..

[3] Mihaljević Z., Matić A., Stupin A., Rašić L., Jukić I., Drenjančević I. (2018). Acute Hyperbaric Oxygenation, Contrary to Intermittent Hyperbaric Oxygenation, Adversely Affects Vasorelaxation in Healthy Sprague-Dawley Rats due to Increased Oxidative Stress. Oxid. Med. Cell. Longev..

[4] Kibel A., Cavka A., Cosic A., Falck J.R., Drenjancevic I. (2012). Effects of hyperbaric oxygenation on vascular reactivity to angiotensin II and angiotensin-(1-7) in rats. Undersea Hyperb. Med..

[5] Kibel A., Novak S., Cosic A., Mihaljevic Z., Falck J.R., Drenjancevic I. (2015). Hyperbaric oxygenation modulates vascular reactivity to angiotensin-(1-7) in diabetic rats: Potential role of epoxyeicosatrienoic acids. Diabetes Vasc. Dis. Res..

[6] Mihaljević Z., Matić A., Stupin A., Frkanec R., Tavčar B., Kelava V., Tartaro Bujak I., Kolobarić N., Kibel A., Drenjančević I. (2020). Arachidonic Acid Metabolites of CYP450 Enzymes and HIF-1α Modulate Endothelium-Dependent Vasorelaxation in Sprague-Dawley Rats under Acute and Intermittent Hyperbaric Oxygenation. Int. J. Mol. Sci..

[7] Carroll M.A., Doumad A.B., Li J., Cheng M.K., Falck J.R., McGiff J.C. (2006). Adenosine2A receptor vasodilation of rat preglomerular microvessels is mediated by EETs that activate the cAMP/PKA pathway. Am. J. Physiol. Renal. Physiol..

[8] Cheng M.K., Doumad A.B., Jiang H., Falck J.R., McGiff J.C., Carroll M.A. (2004). Epoxyeicosatrienoic acids mediate adenosine-induced vasodilation in rat preglomerular microvessels (PGMV) via A2A receptors. Br. J. Pharmacol..

[9] Bruzzese L., Rostain J.C., Née L., Condo J., Mottola G., Adjriou N., Mercier L., Berge-Lefranc J.L., Fromonot J., Kipson N. (2015). Effect of hyperoxic and hyperbaric conditions on the adenosinergic pathway and CD26 expression in rat. J. Appl. Physiol..

[10] Maille B., Lalevée N., Marlinge M., Vahdat J., Mottola G., Degioanni C., De Maria L., Klein V., Thuny F., Franceschi F. (2022). Adenosine and Adenosine Receptors: Advances in Atrial Fibrillation. Biomedicines.

[11] Borea P.A., Gessi S., Merighi S., Vincenzi F., Varani K. (2018). Pharmacology of Adenosine Receptors: The State of the Art. Physiol. Rev..

[12] By Y., Jacquin L., Franceschi F., Durand-Gorde J.M., Condo J., Michelet P., Guieu R., Ruf J. (2012). Fall in oxygen tension of culture medium stimulates the adenosinergic signalling of a human T cell line. Purinergic Signal.

[13] Benson R.M., Minter L.M., Osborne B.A., Granowitz E.V. (2003). Hyperbaric oxygen inhibits stimulus-induced proinflammatory cytokine synthesis by human blood-derived monocyte-macrophages. Clin. Exp. Immunol..

[14] Matic A., Jukic I., Stupin A., Baric L., Mihaljevic Z., Unfirer S., Tartaro Bujak I., Mihaljevic B., Lombard J.H., Drenjancevic I. (2018). High salt intake shifts the mechanisms of flow-induced dilation in the middle cerebral arteries of Sprague-Dawley rats. Am. J. Physiol. Heart Circ. Physiol..

[15] Cosic A., Jukic I., Stupin A., Mihalj M., Mihaljevic Z., Novak S., Vukovic R., Drenjancevic I. (2016). Attenuated flow-induced dilatation of middle cerebral arteries is related to increased vascular oxidative stress in rats on a short-term high salt diet. J. Physiol..

[16] Unfirer S., Mihalj M., Novak S., Kibel A., Cavka A., Mijalevic Z., Gros M., Brizic I., Budimir D., Cosic A. (2016). Hyperbaric oxygenation affects the mechanisms of acetylcholine-induced relaxation in diabetic rats. Undersea Hyperb. Med..

[17] Unfirer S., Drenjančević I. (2011). The mechanisms of vascular reactivity to ACh and serotonin are modulated by hyperbaric oxygen treatment in cerebral resistance arteries of diabetic rats (Pohl U, Sperandio M, editors). J. Vasc. Res..

[18] Unfirer S., Falck J.R., Drenjancevic I. Cytochrome P450-epoxygenase metabolites play role in vasodilation of middle cerebral arteries in response to reduced pO2 in healthy and diabetic rats that underwent hyperbaric oxygenation. Proceedings of the International Union of Physiological Sciences.

[19] Wang Y., Yang J.N., Arner A., Boels P.J., Fredholm B.B. (2010). Adenosine A(1) receptors and vascular reactivity. Acta Physiol..

[20] Bryan P.T., Marshall J.M. (1999). Adenosine receptor subtypes and vasodilatation in rat skeletal muscle during systemic hypoxia: A role for A1 receptors. J. Physiol..

[21] Coney A.M., Marshall J.M. (1998). Role of adenosine and its receptors in the vasodilatation induced in the cerebral cortex of the rat by systemic hypoxia. J. Physiol..

[22] Ngai A.C., Coyne E.F., Meno J.R., West G.A., Winn H.R. (2001). Receptor subtypes mediating adenosine-induced dilation of cerebral arterioles. Am. J. Physiol. Heart Circ. Physiol..

[23] Carroll M.A. (2012). Role of the adenosine(2A) receptor-epoxyeicosatrienoic acid pathway in the development of salt-sensitive hypertension. Prostaglandins Other Lipid Mediat..

[24] Eltzschig H.K., Faigle M., Knapp S., Karhausen J., Ibla J., Rosenberger P., Odegard K.C., Laussen P.C., Thompson L.F., Colgan S.P. (2006). Endothelial catabolism of extracellular adenosine during hypoxia: The role of surface adenosine deaminase and CD26. Blood.

[25] Görlach A. (2005). Control of adenosine transport by hypoxia. Circ. Res..

[26] Bruzzese L., Fromonot J., By Y., Durand-Gorde J.M., Condo J., Kipson N., Guieu R., Fenouillet E., Ruf J. (2014). NF-κB enhances hypoxia-driven T-cell immunosuppression via upregulation of adenosine A(2A) receptors. Cell Signal..

[27] Kunduri S., Dick G., Nayeem M., Mustafa S. (2013). Adenosine A1 receptor signaling inhibits BK channels through a PKCα-dependent mechanism in mouse aortic smooth muscle. Physiol. Rep..

[28] Sharifi-Sanjani M., Zhou X., Asano S., Tilley S., Ledent C., Teng B., Dick G.M., Mustafa S.J. (2013). Interactions between A(2A) adenosine receptors, hydrogen peroxide, and KATP channels in coronary reactive hyperemia. Am. J. Physiol. Heart Circ. Physiol..

[29] DiChiara T.J., Reinhart P.H. (1997). Redox modulation of hslo Ca2+-activated K+ channels. J. Neurosci..

[30] Tang X.D., Daggett H., Hanner M., Garcia M.L., McManus O.B., Brot N., Weissbach H., Heinemann S.H., Hoshi T. (2001). Oxidative regulation of large conductance calcium-activated potassium channels. J. Gen. Physiol..

[31] Carroll M.A., Balazy M., Margiotta P., Huang D.D., Falck J.R., McGiff J.C. (1996). Cytochrome P-450-dependent HETEs: Profile of biological activity and stimulation by vasoactive peptides. Am. J. Physiol..

[32] Koller A., Toth P. (2012). Contribution of flow-dependent vasomotor mechanisms to the autoregulation of cerebral blood flow. J. Vasc. Res..

[33] Ku H.C., Chen W.P., Su M.J. (2013). DPP4 deficiency exerts protective effect against H2O2 induced oxidative stress in isolated cardiomyocytes. PLoS ONE.

[34] Moro P.J., Quilici J., Giorgi R., Cuisset T., By Y., Boussuges A., Jammes Y., Bonnet J.L., Ruf J., Fenouillet E. (2013). Mononuclear cell adenosine deaminase and CD26/dipeptidylpeptidase-IV activities are sensitive markers of reperfusion during percutaneous transluminal angioplasty. Int. J. Cardiol..

[35] Davies J., Karmouty-Quintana H., Le T.T., Chen N.Y., Weng T., Luo F., Molina J., Moorthy B., Blackburn M.R. (2014). Adenosine promotes vascular barrier function in hyperoxic lung injury. Physiol. Rep..

[36] Melani A., Corti F., Cellai L., Vannucchi M.G., Pedata F. (2014). Low doses of the selective adenosine A2A receptor agonist CGS21680 are protective in a rat model of transient cerebral ischemia. Brain Res..

[37] Bryan R.M., Marrelli S.P., Steenberg M.L., Schildmeyer L.A., Johnson T.D. (2001). Effects of luminal shear stress on cerebral arteries and arterioles. Am. J. Physiol. Heart Circ. Physiol..

[38] Toth P., Rozsa B., Springo Z., Doczi T., Koller A. (2011). Isolated human and rat cerebral arteries constrict to increases in flow: Role of 20-HETE and TP receptors. J. Cereb. Blood Flow. Metab..

[39] Kim Y.S., Bogert L.W., Immink R.V., Harms M.P., Colier W.N., van Lieshout J.J. (2011). Effects of aging on the cerebrovascular orthostatic response. Neurobiol. Aging.

[40] Gebremedhin D., Weinberger B., Lourim D., Harder D.R. (2010). Adenosine can mediate its actions through generation of reactive oxygen species. J. Cereb. Blood Flow. Metab..

[41] You J., Golding E.M., Bryan R.M. (2005). Arachidonic acid metabolites, hydrogen peroxide, and EDHF in cerebral arteries. Am. J. Physiol. Heart Circ. Physiol..

[42] Hadanny A., Efrati S. (2020). The Hyperoxic-Hypoxic Paradox. Biomolecules.

[43] Rocco M., D’Itri L., De Bels D., Corazza F., Balestra C. (2014). The “normobaric oxygen paradox”: A new tool for the anesthetist?. Minerva Anestesiol..

[44] Khayat M.T., Nayeem M.A. (2017). The Role of Adenosine A_2A_ Receptor, CYP450s, and PPARs in the Regulation of Vascular Tone. Biomed. Res. Int..

[45] Waypa G.B., Smith K.A., Schumacker P.T. (2016). O2 sensing, mitochondria and ROS signaling: The fog is lifting. Mol. Aspects. Med..

[46] Unfirer S., Kibel A., Drenjancevic-Peric I. (2008). The effect of hyperbaric oxygen therapy on blood vessel function in diabetes mellitus. Med. Hypotheses.

[47] Drenjančević I., Jukić I., Đambić V., Stupin A., Kozina N., Matić A., Šušnjara P., Kibel A., Biljan D., Mihaljević Z. (2025). Variability in flow-induced vasodilation mechanisms in cerebral arteries: The impact of different hyperbaric oxygen protocols. Med. Gas. Res..

[48] Monopoli A., Conti A., Dionisotti S., Casati C., Camaioni E., Cristalli G., Ongini E. (1994). Pharmacology of the highly selective A1 adenosine receptor agonist 2-chloro-N6-cyclopentyladenosine. Arzneimittelforschung.

[49] Monopoli A., Casati C., Lozza G., Forlani A., Ongini E. (1998). Cardiovascular pharmacology of the A2A adenosine receptor antagonist, SCH 58261, in the rat. J. Pharmacol. Exp. Ther..

[50] Conti A., Monopoli A., Gamba M., Borea P.A., Ongini E. (1993). Effects of selective A_1_ and A_2_ adenosine receptor agonists on cardiovascular tissues. Naunyn-Schmiedeberg’s Arch. Pharmacol..

[51] Impellizzeri D., Di Paola R., Esposito E., Mazzon E., Paterniti I., Melani A., Bramanti P., Pedata F., Cuzzocrea S. (2011). CGS 21680, an agonist of the adenosine (A2A) receptor, decreases acute lung inflammation. Eur. J. Pharmacol..

[52] Schneider C.A., Rasband W.S., Eliceiri K.W. (2012). NIH Image to ImageJ: 25 years of image analysis. Nat. Methods.

