# Supplementary material for: The Role of Adenosine A1 and A2a Receptors in Cerebral Blood Vessel Reactivity of Sprague Dawley Rats Exposed to Hyperbaric Oxygenation
Source: Molecules. 2025 Jul 10;30(14):2918. doi: 10.3390/molecules30142918 (PMC12298982; doi:10.3390/molecules30142918)
Supplement: Supplementary file 1 [file molecules-30-02918-s001.zip › molecules-3699884-supplementary.pdf]

Supplement 1.

Figure 1. The response of the MCA of the CTRL, Ac-HBO_2 and In- HBO_2 groups to A1R selective agonist CCPA, applied in stepwise concentrations 10^{-10} - 10^{-5} M. Data are presented as mean ± SD. Significant differences were $^{*\#}p < 0.05$, Ac-HBO_2 compared to CTRL and In-HBO_2, respectively; Two-WayANOVA test was performed.

Table Analyzed	A1 agonist_dose response			
Two-way ANOVA	Ordinary			
Alpha	0.05			
Source of Variation	% of total variation	P value	P value summary	Significant?
Interaction	3.148	0.6024	ns	No
Row Factor	34.83	<0.0001	****	Yes
Column Factor	20.98	<0.0001	****	Yes

TUKEY'S MULTIPLE COMPARISONS TEST	PREDICTED (LS) MEAN DIFF.	95.00% CI OF DIFF.	SIGNIFICANT?	SUMMARY	ADJUSTED P VALUE
**ROW 1**					
**CTRL A1R VS. AC-HBO_2 A1R**	2.715	-5.877 to 11.31	No	ns	0.6877
**CTRL A1R VS. IN-HBO_2 A1R**	-1.682	-10.27 to 6.909	No	ns	0.9835
**AC-HBO_2 A1R VS. IN-HBO_2 A1R**	-4.397	-12.99 to 4.195	No	ns	0.5784
**ROW 2**					
**CTRL A1R VS. AC-HBO_2 A1R**	6.529	-2.062 to 15.12	No	ns	0.1399
**CTRL A1R VS. IN-HBO_2 A1R**	-2.075	-10.67 to 6.517	No	ns	0.9958

**AC-HBO$_2$ A1R VS. IN-HBO$_2$ A1R**	-8.604	-17.20 to -0.01273	No	ns	0.1170
**ROW 3**					
**CTRL A1R VS. AC-HBO$_2$ A1R**	5.293	-3.298 to 13.88	No	ns	0.1877
**CTRL A1R VS. IN-HBO$_2$ A1R**	-5.091	-13.68 to 3.501	No	ns	0.7537
**AC-HBO$_2$ A1R VS. IN-HBO$_2$ A1R**	-10.38	-18.98 to -1.792	Yes	*	0.0388
**ROW 4**					
**CTRL A1R VS. AC-HBO$_2$ A1R**	4.221	-4.370 to 12.81	No	ns	0.2463
**CTRL A1R VS. IN-HBO$_2$ A1R**	-6.491	-15.08 to 2.100	No	ns	0.5648
**AC-HBO$_2$ A1R VS. IN-HBO$_2$ A1R**	-10.71	-19.30 to -2.120	Yes	*	0.0262
**ROW 5**					
**CTRL A1R VS. AC-HBO$_2$ A1R**	7.304	-1.287 to 15.90	Yes	*	0.0348
**CTRL A1R VS. IN-HBO$_2$ A1R**	-8.709	-17.30 to -0.1173	No	ns	0.3604
**AC-HBO$_2$ A1R VS. IN-HBO$_2$ A1R**	-16.01	-24.60 to -7.422	Yes	***	0.0005
**ROW 6**					
**CTRL A1R VS. AC-HBO$_2$ A1R**	6.599	-1.992 to 15.19	Yes	*	0.0368
**CTRL A1R VS. IN-HBO$_2$ A1R**	-9.082	-17.67 to -0.4904	No	ns	0.4203

**AC-HBO_2 A1R VS. IN-HBO_2 A1R**	-15.68	-24.27 to -7.090	Yes	***	0.0008

Figure 2. The response of the MCA of the CTRL, rats exposed to Ac-HBO$_2$ and In- HBO$_2$, to the application of the A2aR agonist CGS-21680 in stepwise concentrations 10^{-10} - 10^{-5} M. Data are presented as mean ± SD. Significant differences were *#$p < 0.05$, Ac-HBO$_2$ compared to CTRL and In-HBO$_2$, respectively; Two-WayANOVA test was performed.

Table Analyzed	A2 agonist_dose response			
Two-way ANOVA	Ordinary			
Alpha	0.05			
Source of Variation	% of total variation	P value	P value summary	Significant?
Interaction	9.117	0.0709	ns	No
Row Factor	29.89	<0.0001	****	Yes

TUKEY'S MULTIPLE COMPARISONS TEST	PREDICTED (LS) MEAN DIFF.	95.00% CI OF DIFF.	SIGNIFICANT?	SUMMARY	ADJUSTED P VALUE
**ROW 1**					
**CTRL A2AR VS. AC-HBO$_2$ A2AR**	-0.5365	-11.27 to 10.20	No	ns	0.9948
**CTRL A2AR VS. IN-HBO$_2$ A2AR**	-1.852	-12.59 to 8.881	No	ns	0.9739
**AC-HBO$_2$ A2AR VS. IN-HBO$_2$ A2AR**	-1.316	-12.05 to 9.418	No	ns	0.9919
**ROW 2**					
**CTRL A2AR VS. AC-HBO$_2$ A2AR**	-1.756	-12.49 to 8.977	No	ns	0.9381
**CTRL A2AR VS. IN-HBO$_2$ A2AR**	-1.974	-12.71 to 8.759	No	ns	0.9971
**AC-HBO$_2$ A2AR VS. IN-HBO$_2$ A2AR**	-0.2180	-10.95 to 10.52	No	ns	0.9101

**ROW 3**					
**CTRL A2AR VS. AC-HBO_2 A2AR**	-0.8272	-11.56 to 9.906	No	ns	0.9793
**CTRL A2AR VS. IN-HBO_2 A2AR**	-2.947	-13.68 to 7.787	No	ns	0.9987
**AC-HBO_2 A2AR VS. IN-HBO_2 A2AR**	-2.120	-12.85 to 8.614	No	ns	0.9679
**ROW 4**					
**CTRL A2AR VS. AC-HBO_2 A2AR**	6.487	-4.246 to 17.22	No	ns	0.1909
**CTRL A2AR VS. IN-HBO_2 A2AR**	-4.228	-14.96 to 6.506	No	ns	0.9997
**AC-HBO_2 A2AR VS IN-HBO_2 A2AR**	-10.72	-21.45 to 0.01836	No	ns	0.1997
**ROW 5**					
**CTRL A2AR VS. AC-HBO_2 A2AR**	10.20	-0.5352 to 20.93	Yes	*	0.0135
**CTRL A2AR VS. IN-HBO_2 A2AR**	-3.777	-14.51 to 6.956	No	ns	0.9293
**AC-HBO_2 A2AR VS. IN-HBO_2 A2AR**	-13.98	-24.71 to -3.242	Yes	*	0.0362
**ROW 6**					
**CTRL A2AR VS. AC-HBO_2 A2AR**	13.17	2.433 to 23.90	Yes	**	0.0015
**CTRL A2AR VS. IN-HBO_2 A2AR**	-4.616	-15.35 to 6.118	No	ns	0.9293
**AC-HBO_2 A2AR VS. IN-HBO_2 A2AR**	-17.78	-28.52 to -7.049	Yes	**	0.0049

Figure 3. Flow-induced dilation (FID), acetylcholine-induced response (ACh), and sodium-nitroprusside (SNP)-induced response of middle cerebral arteries (MCA). FID is presented as the percentage of dilatation (%) of MCA in response to stepwise increases in the pressure gradient (Δ10-Δ100 mmHg) compared to baseline (no flow condition, Δ0 mmHg) (a). ACh (b) and SNP (c) were tested under no flow conditions (at 80 mmHg). Data are presented as mean ± SD. Significant differences were $^{*\#}p < 0.05$, Ac-HBO_2 compared to CTRL and In-HBO_2, respectively. Two-WayANOVA (a) or One-WayANOVA test (b and c) was performed, respectively.

Figure 3a

Table Analyzed	Basal (FID)			
Two-way ANOVA	Ordinary			
Alpha	0.05			
Source of Variation	% of total variation	P value	P value summary	Significant?
Interaction	1.765	0.1035	ns	No
Row Factor	38.63	<0.0001	****	Yes

TUKEY'S MULTIPLE COMPARISONS TEST	PREDICTED (LS) MEAN DIFF.	95.00% CI OF DIFF.	SIGNIFICANT?	SUMMARY	ADJUSTED P VALUE
**ROW 1**					
**CTRL VS. AC-HBO_2**	1.187	-2.621 to 4.995	No	ns	0.7440
**CTRL VS. IN-HBO_2**	0.1771	-3.596 to 3.950	No	ns	0.9933
**AC-HBO_2 VS. IN-HBO_2**	-1.010	-4.818 to 2.798	No	ns	0.8072
**ROW 2**					
**CTRL VS. AC-HBO_2**	2.324	-1.449 to 6.097	No	ns	0.3171
**CTRL VS. IN-HBO_2**	-0.2704	-4.044 to 3.503	No	ns	0.9845
**AC-HBO_2 VS. IN-HBO_2**	-2.595	-6.368 to 1.179	No	ns	0.2396

**ROW 3**					
**CTRL VS. AC-HBO$_2$**	2.339	-1.434 to 6.112	No	ns	0.3126
**CTRL VS. IN-HBO$_2$**	-1.619	-5.392 to 2.154	No	ns	0.5716
**AC-HBO$_2$ VS. IN-HBO$_2$**	-3.958	-7.731 to -0.1850	Yes	*	0.0372
**ROW 4**					
**CTRL VS. AC-HBO$_2$**	4.537	0.7636 to 8.310	Yes	*	0.0136
**CTRL VS. IN-HBO$_2$**	-0.9479	-4.721 to 2.825	No	ns	0.8252
**AC-HBO$_2$ VS. IN-HBO$_2$**	-5.485	-9.258 to -1.711	Yes	**	0.0020
**ROW 5**					
**CTRL VS. AC-HBO$_2$**	3.950	0.1765 to 7.723	Yes	*	0.0377
**CTRL VS. IN-HBO$_2$**	-3.289	-7.062 to 0.4839	No	ns	0.1017
**AC-HBO$_2$ VS. IN-HBO$_2$**	-7.239	-11.01 to -3.466	Yes	****	<0.0001
**ROW 6**					
**CTRL VS. AC-HBO$_2$**	4.930	1.156 to 8.703	Yes	**	0.0064
**CTRL VS. IN-HBO$_2$**	-3.498	-7.271 to 0.2757	No	ns	0.0758
**AC-HBO$_2$ VS. IN-HBO$_2$**	-8.427	-12.20 to -4.654	Yes	****	<0.0001

Figure 3b

Table Analyzed	ACh

Data sets analyzed	A-C
ANOVA summary	
F	13.97
P value	0.0002
P value summary	***
Significant diff. among means (P < 0.05)?	Yes
R square	0.6083

TUKEY'S MULTIPLE COMPARISONS TEST	MEAN DIFF.	95.00% CI OF DIFF.	SIGNIFICANT?	SUMMARY	ADJUSTED P VALUE
**CTRL VS. AC-HBO$_2$**	8.710	3.252 to 14.17	Yes	**	0.0020
**CTRL VS. IN-HBO$_2$**	-1.887	-7.345 to 3.571	No	ns	0.6579
**AC-HBO$_2$ VS. IN-HBO$_2$**	-10.60	-16.05 to -5.139	Yes	***	0.0003

Figure 3c

Table Analyzed	SNP
Data sets analyzed	A-C
ANOVA summary	
F	0.6001
P value	0.5593
P value summary	ns
Significant diff. among means (P < 0.05)?	No
R square	0.06251

TUKEY'S MULTIPLE COMPARISONS TEST	MEAN DIFF.	95.00% CI OF DIFF.	SIGNIFICANT?	SUMMARY	ADJUSTED P VALUE

**CTRL VS. AC-HBO_2**	-0.6086	-6.298 to 5.081	No	ns	0.9598
**CTRL VS. IN-HBO_2**	-2.353	-8.043 to 3.337	No	ns	0.5529
**AC-HBO_2 VS. IN-HBO_2**	-1.744	-7.434 to 3.946	No	ns	0.7183

Figure 4. FID of MCA in the presence of A1R and A2aR agonists (CCPA, 10^{-6} M and CGS-21680, 10^{-6} M, respectively) or antagonists (DPCPX, 10^{-6} M and SCH-58261, 10^{-6} M, respectively) in the CTRL (a and b), Ac-HBO_2 (c and d) and In-HBO_2 (e and f) groups of rats. Results are presented as mean ± SD; *p < 0.05 compared to baseline, Two-WayANOVA test was performed.

Figure 4a

Table Analyzed	CTRL+agonists (FID)			
Two-way ANOVA	Ordinary			
Alpha	0.05			
Source of Variation	% of total variation	P value	P value summary	Significant?
Interaction	1.174	0.9436	ns	No
Row Factor	16.23	<0.0001	****	Yes
Column Factor	1.049	0.1662	ns	No

TUKEY'S MULTIPLE COMPARISONS TEST	MEAN DIFF.	95.00% CI OF DIFF.	SIGNIFICANT?	SUMMARY	ADJUSTED P VALUE
**ROW 1**					
**CTRL VS. CTRL+A1 AGONIST**	0.5398	-6.560 to 7.639	No	ns	0.9824
**CTRL VS. CTRL+A2A AGONIST**	-0.3007	-7.400 to 6.799	No	ns	0.9945
**CTRL+A1 AGONIST VS. CTRL+A2A AGONIST**	-0.8405	-9.821 to 8.140	No	ns	0.9735
**ROW 2**					
**CTRL VS. CTRL+A1 AGONIST**	-0.2697	-7.369 to 6.830	No	ns	0.9956
**CTRL VS. CTRL+A2A AGONIST**	1.827	-5.273 to 8.926	No	ns	0.8165
**CTRL+A1 AGONIST VS. CTRL+A2A AGONIST**	2.096	-6.884 to 11.08	No	ns	0.8462
**ROW 3**					

**CTRL VS. CTRL+A1 AGONIST**	-0.7603	-7.860 to 6.339	No	ns	0.9654
**CTRL VS. CTRL+A2A AGONIST**	1.632	-5.467 to 8.732	No	ns	0.8505
**CTRL+A1 AGONIST VS. CTRL+A2A AGONIST**	2.393	-6.587 to 11.37	No	ns	0.8046
**ROW 4**					
**CTRL VS. CTRL+A1 AGONIST**	3.036	-4.063 to 10.14	No	ns	0.5721
**CTRL VS. CTRL+A2A AGONIST**	0.8443	-6.255 to 7.944	No	ns	0.9576
**CTRL+A1 AGONIST VS. CTRL+A2A AGONIST**	-2.192	-11.17 to 6.788	No	ns	0.8332
**ROW 5**					
**CTRL VS. CTRL+A1 AGONIST**	1.790	-5.310 to 8.889	No	ns	0.8232
**CTRL VS. CTRL+A2A AGONIST**	2.911	-4.188 to 10.01	No	ns	0.5983
**CTRL+A1 AGONIST VS. CTRL+A2A AGONIST**	1.122	-7.858 to 10.10	No	ns	0.9533
**ROW 6**					
**CTRL VS. CTRL+A1 AGONIST**	4.013	-3.087 to 11.11	No	ns	0.3782
**CTRL VS. CTRL+A2A AGONIST**	5.794	-1.305 to 12.89	No	ns	0.1339
**CTRL+A1 AGONIST VS. CTRL+A2A AGONIST**	1.781	-7.199 to 10.76	No	ns	0.8864

Figure 4b

Table Analyzed	CTRL+antagonists (FID)
Two-way ANOVA	Ordinary
Alpha	0.05

Source of Variation	% of total variation	P value	P value summary	Significant?
Interaction	0.4155	0.9982	ns	No
Row Factor	20.00	<0.0001	****	Yes
Column Factor	7.166	<0.0001	****	Yes

TUKEY'S MULTIPLE COMPARISONS TEST	MEAN DIFF.	95.00% CI OF DIFF.	SIGNIFICANT ?	SUMMARY	ADJUSTED P VALUE
**ROW 1**					
**CTRL VS. CTRL+A1 ANTAGONIST**	3.328	-3.093 to 9.749	No	ns	0.4411
**CTRL VS. CTRL+A2A ANTAGONIST**	3.725	-2.696 to 10.15	No	ns	0.3592
**CTRL+A1 ANTAGONIST VS. CTRL+A2A ANTAGONIST**	0.3971	-7.725 to 8.519	No	ns	0.9927
**ROW 2**					
**CTRL VS. CTRL+A1 ANTAGONIST**	3.254	-3.167 to 9.675	No	ns	0.4571
**CTRL VS. CTRL+A2A ANTAGONIST**	2.823	-3.599 to 9.244	No	ns	0.5544
**CTRL+A1 ANTAGONIST VS. CTRL+A2A ANTAGONIST**	-0.4314	-8.553 to 7.691	No	ns	0.9914
**ROW 3**					
**CTRL VS. CTRL+A1 ANTAGONIST**	3.988	-2.433 to 10.41	No	ns	0.3097
**CTRL VS. CTRL+A2A ANTAGONIST**	5.085	-1.336 to 11.51	No	ns	0.1503
**CTRL+A1 ANTAGONIST VS. CTRL+A2A ANTAGONIST**	1.097	-7.025 to 9.219	No	ns	0.9456

**ROW 4**					
**CTRL VS. CTRL+A1 ANTAGONIST**	5.270	-1.151 to 11.69	No	ns	0.1309
**CTRL VS. CTRL+A2A ANTAGONIST**	6.537	0.1161 to 12.96	No	ns	0.0549
**CTRL+A1 ANTAGONIST VS. CTRL+A2A ANTAGONIST**	1.267	-6.855 to 9.389	No	ns	0.9281
**ROW 5**					
**CTRL VS. CTRL+A1 ANTAGONIST**	4.660	-1.761 to 11.08	No	ns	0.2029
**CTRL VS. CTRL+A2A ANTAGONIST**	6.097	-0.3239 to 12.52	No	ns	0.0667
**CTRL+A1 ANTAGONIST VS. CTRL+A2A ANTAGONIST**	1.437	-6.685 to 9.559	No	ns	0.9085
**ROW 6**					
**CTRL VS. CTRL+A1 ANTAGONIST**	5.264	-1.157 to 11.68	No	ns	0.1315
**CTRL VS. CTRL+A2A ANTAGONIST**	5.115	-1.306 to 11.54	No	ns	0.1470
**CTRL+A1 ANTAGONIST VS. CTRL+A2A ANTAGONIST**	-0.1486	-8.271 to 7.973	No	ns	0.9990

Figure 4c

Table Analyzed	Ac-HBO$_2$ + agonists (FID)			
Two-way ANOVA	Ordinary			
Alpha	0.05			
Source of Variation	% of total variation	P value	P value summary	Significant?
Interaction	1.894	0.7938	ns	No
Row Factor	10.68	<0.0001	****	Yes
Column Factor	4.522	0.0007	***	Yes

TUKEY'S MULTIPLE COMPARISONS TEST	MEAN DIFF.	95.00% CI OF DIFF.	SIGNIFICANT?	SUMMARY	ADJUSTED P VALUE
**ROW 1**					
**AC-HBO$_2$ VS. AC-HBO$_2$ + A1 AGONIST**	0.3292	-5.525 to 6.183	No	ns	0.9903
**AC-HBO$_2$ VS. AC-HBO$_2$ + A2A AGONIST**	-0.01365	-5.868 to 5.841	No	ns	>0.9999
**AC-HBO$_2$ + A1 AGONIST VS. AC-HBO$_2$ + A2A AGONIST**	-0.3429	-7.721 to 7.035	No	ns	0.9934
**ROW 2**					
**AC-HBO$_2$ VS. AC-HBO$_2$ + A1 AGONIST**	1.284	-4.549 to 7.117	No	ns	0.8620
**AC-HBO$_2$ VS. AC-HBO$_2$ + A2A AGONIST**	-0.09464	-5.927 to 5.738	No	ns	0.9992
**AC-HBO$_2$ + A1 AGONIST VS. AC-HBO$_2$ + A2A AGONIST**	-1.379	-8.756 to 5.999	No	ns	0.8985
**ROW 3**					
**AC-HBO$_2$ VS. AC-HBO$_2$ + A1 AGONIST**	4.458	-1.375 to 10.29	No	ns	0.1709
**AC-HBO$_2$ VS. AC-HBO$_2$ + A2A AGONIST**	1.236	-4.596 to 7.069	No	ns	0.8714
**AC-HBO$_2$ + A1 AGONIST VS. AC-HBO$_2$ + A2A AGONIST**	-3.221	-10.60 to 4.156	No	ns	0.5587
**ROW 4**					

**$AC\text{-}HBO_2$ VS. $AC\text{-}HBO_2$ + A1 AGONIST**	6.468	0.6349 to 12.30	No	ns	0.0756
**$AC\text{-}HBO_2$ VS. $AC\text{-}HBO_2$ + A2A AGONIST**	2.768	-3.065 to 8.600	No	ns	0.5032
**$AC\text{-}HBO_2$ + A1 AGONIST VS. $AC\text{-}HBO_2$ + A2A AGONIST**	-3.700	-11.08 to 3.678	No	ns	0.4645
**ROW 5**					
**$AC\text{-}HBO_2$ VS. $AC\text{-}HBO_2$ + A1 AGONIST**	5.605	-0.2280 to 11.44	No	ns	0.0626
**$AC\text{-}HBO_2$ VS. $AC\text{-}HBO_2$ + A2A AGONIST**	3.790	-2.042 to 9.623	No	ns	0.2774
**$AC\text{-}HBO_2$ + A1 AGONIST VS. $AC\text{-}HBO_2$ + A2A AGONIST**	-1.814	-9.192 to 5.563	No	ns	0.8309
**ROW 6**					
**$AC\text{-}HBO_2$ VS. $AC\text{-}HBO_2$ + A1 AGONIST**	4.271	-1.561 to 10.10	No	ns	0.1971
**$AC\text{-}HBO_2$ VS. $AC\text{-}HBO_2$ + A2A AGONIST**	3.301	-2.531 to 9.134	No	ns	0.3772
**$AC\text{-}HBO_2$ + A1 AGONIST VS. $AC\text{-}HBO_2$ + A2A AGONIST**	-0.9700	-8.348 to 6.408	No	ns	0.9484

Figure 4d

Table Analyzed	$Ac\text{-}HBO_2$+antagonists(FID)			
Two-way ANOVA	Ordinary			
Alpha	0.05			
Source of Variation	% of total variation	P value	P value summary	Significant?
Interaction	0.1649	>0.9999	ns	No
Row Factor	18.00	<0.0001	****	Yes
Column Factor	1.105	0.1731	ns	No

TUKEY'S MULTIPLE COMPARISONS TEST	MEAN DIFF.	95.00% CI OF DIFF.	SIGNIFICANT?	SUMMARY	ADJUSTED P VALUE
**ROW 1**					
**$AC-HBO_2$ VS. $AC-HBO_2$ + A1 ANTAGONIST**	-1.012	-7.630 to 5.605	No	ns	0.9308
**$AC-HBO_2$ VS. $AC-HBO_2$ + A2A ANTAGONIST**	2.005	-4.613 to 8.623	No	ns	0.7551
**$AC-HBO_2$ + A1 ANTAGONIST VS. $AC-HBO_2$ + A2A ANTAGONIST**	3.017	-5.323 to 11.36	No	ns	0.6702
**ROW 2**					
**$AC-HBO_2$ VS. $AC-HBO_2$ + A1 ANTAGONIST**	-1.723	-8.317 to 4.870	No	ns	0.8112
**$AC-HBO_2$ VS. $AC-HBO_2$ + A2A ANTAGONIST**	0.6011	-5.992 to 7.194	No	ns	0.9748
**$AC-HBO_2$ + A1 ANTAGONIST VS. $AC-HBO_2$ + A2A ANTAGONIST**	2.324	-6.016 to 10.66	No	ns	0.7884
**ROW 3**					
**$AC-HBO_2$ VS. $AC-HBO_2$ + A1 ANTAGONIST**	-1.158	-7.751 to 5.435	No	ns	0.9098
**$AC-HBO_2$ VS. $AC-HBO_2$ + A2A ANTAGONIST**	2.539	-4.054 to 9.133	No	ns	0.6355
**$AC-HBO_2$ + A1 ANTAGONIST VS. $AC-HBO_2$ + A2A ANTAGONIST**	3.697	-4.643 to 12.04	No	ns	0.5489
**ROW 4**					
**$AC-HBO_2$ VS. $AC-HBO_2$ + A1 ANTAGONIST**	-1.301	-7.894 to 5.292	No	ns	0.8875
**$AC-HBO_2$ VS. $AC-HBO_2$ + A2A ANTAGONIST**	1.016	-5.577 to 7.609	No	ns	0.9298
**$AC-HBO_2$ + A1 ANTAGONIST VS. $AC-HBO_2$ + A2A ANTAGONIST**	2.317	-6.023 to 10.66	No	ns	0.7895
**ROW 5**					

**AC-HBO$_2$ VS. AC-HBO$_2$ + A1 ANTAGONIST**	-0.7282	-7.322 to 5.865	No	ns	0.9633
**AC-HBO$_2$ VS. AC-HBO$_2$ + A2A ANTAGONIST**	1.475	-5.119 to 8.068	No	ns	0.8579
**AC-HBO$_2$ + A1 ANTAGONIST VS. AC-HBO$_2$ + A2A ANTAGONIST**	2.203	-6.137 to 10.54	No	ns	0.8076
**ROW 6**					
**AC-HBO$_2$ VS. AC-HBO$_2$ + A1 ANTAGONIST**	-2.179	-8.772 to 4.415	No	ns	0.7161
**AC-HBO$_2$ VS. AC-HBO$_2$ + A2A ANTAGONIST**	0.5457	-6.048 to 7.139	No	ns	0.9792
**AC-HBO$_2$ + A1 ANTAGONIST VS. AC-HBO$_2$ + A2A ANTAGONIST**	2.724	-5.616 to 11.06	No	ns	0.7215

Figure 4e

Table Analyzed	In-HBO$_2$+agonists (FID)			
Two-way ANOVA	Ordinary			
Alpha	0.05			
Source of Variation	% of total variation	P value	P value summary	Significant?
Interaction	2.523	0.2183	ns	No
Row Factor	23.98	<0.0001	****	Yes
Column Factor	5.317	<0.0001	****	Yes

TUKEY'S MULTIPLE COMPARISONS TEST	MEAN DIFF.	95.00% CI OF DIFF.	SIGNIFICANT?	SUMMARY	ADJUSTED P VALUE
**ROW 1**					
**IN-HBO$_2$ VS. IN-HBO$_2$ + A1 AGONIST**	-0.3693	-5.941 to 5.203	No	ns	0.9866
**IN-HBO$_2$ VS. IN-HBO2 + A2A AGONIST**	0.6807	-4.891 to 6.253	No	ns	0.9553
**IN-HBO$_2$ + A1 AGONIST VS. IN-HBO$_2$ + A2A AGONIST**	1.050	-5.998 to 8.098	No	ns	0.9342

**ROW 2**					
**IN-HBO$_2$ VS. IN-HBO$_2$ + A1 AGONIST**	-1.249	-6.821 to 4.323	No	ns	0.8574
**IN-HBO$_2$ VS. IN-HBO2 + A2A AGONIST**	2.330	-3.242 to 7.902	No	ns	0.5862
**IN-HBO$_2$ + A1 AGONIST VS. IN-HBO$_2$ + A2A AGONIST**	3.579	-3.470 to 10.63	No	ns	0.4558
**ROW 3**					
**IN-HBO$_2$ VS. IN-HBO$_2$ + A1 AGONIST**	-1.601	-7.173 to 3.971	No	ns	0.7767
**IN-HBO$_2$ VS. IN-HBO2 + A2A AGONIST**	3.430	-2.142 to 9.002	No	ns	0.3161
**IN-HBO$_2$ + A1 AGONIST VS. IN-HBO$_2$ + A2A AGONIST**	5.031	-2.017 to 12.08	No	ns	0.2135
**ROW 4**					
**IN-HBO$_2$ VS. IN-HBO$_2$ + A1 AGONIST**	0.4021	-5.170 to 5.974	No	ns	0.9842
**IN-HBO$_2$ VS. IN-HBO2 + A2A AGONIST**	6.785	1.213 to 12.36	Yes	*	0.0123
**IN-HBO$_2$ + A1 AGONIST VS. IN-HBO$_2$ + A2A AGONIST**	6.383	-0.6653 to 13.43	No	ns	0.0848
**ROW 5**					
**IN-HBO$_2$ VS. IN-HBO$_2$ + A1 AGONIST**	3.119	-2.453 to 8.691	No	ns	0.3852
**IN-HBO$_2$ VS. IN-HBO2 + A2A AGONIST**	6.675	1.103 to 12.25	Yes	*	0.0141
**IN-HBO$_2$ + A1 AGONIST VS. IN-HBO$_2$ + A2A AGONIST**	3.556	-3.492 to 10.60	No	ns	0.4603
**ROW 6**					
**IN-HBO$_2$ VS. IN-HBO$_2$ + A1 AGONIST**	1.856	-3.716 to 7.428	No	ns	0.7123

**IN-HBO$_2$ VS. IN-HBO2 + A2A AGONIST**	10.40	4.832 to 15.98	Yes	****	<0.0001
**IN-HBO$_2$ + A1 AGONIST VS. IN-HBO$_2$ + A2A AGONIST**	8.549	1.500 to 15.60	Yes	*	0.0128

Figure 4f

Table Analyzed	In-HBO$_2$+antagonists (FID)			
Two-way ANOVA	Ordinary			
Alpha	0.05			
Source of Variation	% of total variation	P value	P value summary	Significant?
Interaction	0.9946	0.8508	ns	No
Row Factor	29.63	<0.0001	****	Yes
Column Factor	3.250	0.0002	***	Yes

TUKEY'S MULTIPLE COMPARISONS TEST	MEAN DIFF.	95.00% CI OF DIFF.	SIGNIFICANT?	SUMMARY	ADJUSTED P VALUE
**ROW 1**					
**IN-HBO$_2$ VS. IN-HBO$_2$ + A1 ANTAGONIST**	0.3350	-4.957 to 5.627	No	ns	0.9878
**IN-HBO$_2$ VS. IN-HBO$_2$ + A2A ANTAGONIST**	0.3021	-4.989 to 5.594	No	ns	0.9901
**IN-HBO$_2$ + A1 ANTAGONIST VS. IN-HBO$_2$ + A2A ANTAGONIST**	-0.03286	-6.726 to 6.661	No	ns	>0.9999
**ROW 2**					
**IN-HBO$_2$ VS. IN-HBO$_2$ + A1 ANTAGONIST**	1.330	-3.962 to 6.622	No	ns	0.8241
**IN-HBO$_2$ VS. IN-HBO$_2$ + A2A ANTAGONIST**	2.424	-2.867 to 7.716	No	ns	0.5270
**IN-HBO$_2$ + A1 ANTAGONIST VS. IN-HBO$_2$ + A2A ANTAGONIST**	1.094	-5.599 to 7.788	No	ns	0.9213
**ROW 3**					

**IN-HBO$_2$ VS. IN-HBO$_2$ + A1 ANTAGONIST**	1.986	-3.306 to 7.278	No	ns	0.6501
**IN-HBO$_2$ VS. IN-HBO$_2$ + A2A ANTAGONIST**	3.242	-2.050 to 8.533	No	ns	0.3196
**IN-HBO$_2$ + A1 ANTAGONIST VS. IN-HBO$_2$ + A2A ANTAGONIST**	1.256	-5.438 to 7.949	No	ns	0.8978
**ROW 4**					
**IN-HBO$_2$ VS. IN-HBO$_2$ + A1 ANTAGONIST**	3.598	-1.694 to 8.889	No	ns	0.2461
**IN-HBO$_2$ VS. IN-HBO$_2$ + A2A ANTAGONIST**	4.155	-1.137 to 9.447	No	ns	0.1552
**IN-HBO$_2$ + A1 ANTAGONIST VS. IN-HBO$_2$ + A2A ANTAGONIST**	0.5571	-6.136 to 7.251	No	ns	0.9790
**ROW 5**					
**IN-HBO$_2$ VS. IN-HBO$_2$ + A1 ANTAGONIST**	4.319	-0.9723 to 9.611	No	ns	0.1338
**IN-HBO$_2$ VS. IN-HBO$_2$ + A2A ANTAGONIST**	4.551	-0.7409 to 9.842	No	ns	0.1077
**IN-HBO$_2$ + A1 ANTAGONIST VS. IN-HBO$_2$ + A2A ANTAGONIST**	0.2314	-6.462 to 6.925	No	ns	0.9963
**ROW 6**					
**IN-HBO$_2$ VS. IN-HBO$_2$ + A1 ANTAGONIST**	5.550	0.2584 to 10.84	No	ns	0.0873
**IN-HBO$_2$ VS. IN-HBO$_2$ + A2A ANTAGONIST**	4.327	-0.9644 to 9.619	No	ns	0.1329
**IN-HBO$_2$ + A1 ANTAGONIST VS. IN-HBO$_2$ + A2A ANTAGONIST**	-1.223	-7.916 to 5.471	No	ns	0.9028

Figure 5. Hypoxia-induced dilation in the MCA of the CTRL, Ac-HBO_2 and In-HBO_2 groups without (a) and with A1R agonist – CCPA (b), A2aR agonist – CGS-21680 (c), A1R antagonist – DPCPX (d) and A2aR antagonist – SCH-58261 (e). Results are presented as mean ± SD; number of rats; $^{*\#}$ $p < 0.05$, Ac-HBO_2 compared to CTRL and In-HBO_2, respectively; one-way ANOVA test. Data are presented as mean ± SD.

Figure 5a

Table Analyzed	basal_hypoxia
Data sets analyzed	A-C
ANOVA summary	
F	6.906
P value	0.0018
P value summary	**
Significant diff. among means (P < 0.05)?	Yes
R square	0.1628

HOLM-SIDAK'S MULTIPLE COMPARISONS TEST	MEAN DIFF.	SIGNIFICANT?	SUMMARY	ADJUSTED P VALUE
**AC-HBO_2 BASELINE VS. CTRL BASELINE**	6.602	Yes	*	0.0330
**IN-HBO_2 BASELINE VS. CTRL BASELINE**	-3.228	No	ns	0.2296
**IN-HBO_2 BASELINE VS. AC-HBO_2 BASELINE**	-9.830	Yes	**	0.0015

Figure 5b

Table Analyzed	A1 agonist through groups (hypoxia)
Data sets analyzed	A-F
ANOVA summary	
F	4.714
P value	0.0007
P value summary	***

Significant diff. among means ($P < 0.05$)?	Yes
R square	0.2094

TUKEY'S MULTIPLE COMPARISONS TEST	MEAN DIFF.	95.00% CI OF DIFF.	SIGNIFICANT ?	SUMMARY	ADJUSTED P VALUE
**CTRL BASELINE VS. CTRL + A1 AGONIST**	6.774	-5.063 to 18.61	No	ns	0.5570
**CTRL BASELINE VS. AC-HBO$_2$ BASELINE**	6.602	-1.309 to 14.51	Yes	*	0.0330
**CTRL BASELINE VS. AC-HBO$_2$ + A1 AGONIST**	11.50	-0.3360 to 23.34	No	ns	0.0619
**CTRL BASELINE VS. IN-HBO$_2$ BASELINE**	-3.228	-11.06 to 4.602	No	ns	0.2296
**CTRL BASELINE VS. IN-HBO$_2$ + A1 AGONIST**	-0.5899	-12.43 to 11.25	No	ns	>0.9999
**CTRL + A1 AGONIST VS. AC-HBO$_2$ BASELINE**	-0.1721	-12.06 to 11.72	No	ns	>0.9999
**CTRL + A1 AGONIST VS. AC-HBO$_2$ + A1 AGONIST**	4.727	-10.07 to 19.52	No	ns	0.9376
**CTRL + A1 AGONIST VS. IN-HBO$_2$ BASELINE**	-10.00	-21.84 to 1.835	No	ns	0.1471
**CTRL + A1 AGONIST VS. IN-HBO$_2$ + A1 AGONIST**	-7.364	-22.16 to 7.433	No	ns	0.6967
**AC-HBO$_2$ BASELINE VS. AC-HBO$_2$ + A1 AGONIST**	4.899	-6.992 to 16.79	No	ns	0.8357
**AC-HBO$_2$ BASELINE VS. IN-HBO$_2$ BASELINE**	-9.830	-17.74 to -1.919	Yes	**	0.0015
**AC-HBO$_2$ BASELINE VS. IN-HBO$_2$ A1 AGONIST**	-7.192	-19.08 to 4.699	No	ns	0.4955
**AC-HBO$_2$ + A1 AGONIST VS. IN-HBO$_2$ BASELINE**	-14.73	-26.57 to -2.892	Yes	**	0.0062
**AC-HBO$_2$ + A1 AGONIST VS. IN-HBO$_2$ + A1 AGONIST**	-12.09	-26.89 to 2.705	No	ns	0.1745

**IN-HBO$_2$ BASELINE VS. IN-HBO$_2$ + A1 AGONIST**	2.638	-9.199 to 14.48	No	ns	0.9868

Figure 5c

Table Analyzed	A2a agonist through groups (hypoxia)
Data sets analyzed	A-F
ANOVA summary	
F	4.326
P value	0.0014
P value summary	**
Significant diff. among means (P < 0.05)?	Yes
R square	0.1955

TUKEY'S MULTIPLE COMPARISONS TEST	MEAN DIFF.	95.00% CI OF DIFF.	SIGNIFICANT ?	SUMMARY	ADJUSTED P VALUE
**CTRL BASELINE VS. CTRL+A2A AGONIST**	1.046	-10.33 to 12.42	No	ns	0.9998
**CTRL BASELINE VS. AC-HBO$_2$ BASELINE**	6.602	-1.309 to 14.51	Yes	*	0.0330
**CTRL BASELINE VS. AC-HBO$_2$ +A2A AGONIST**	9.066	-2.312 to 20.44	No	ns	0.1967
**CTRL BASELINE VS. IN-HBO$_2$ BASELINE**	-3.228	-10.75 to 4.297	No	ns	0.8111
**CTRL BASELINE VS. IN-HBO$_2$ +A2A AGONIST**	-3.370	-14.75 to 8.007	No	ns	0.9543
**CTRL+A2A AGONIST VS. AC-HBO$_2$ BASELINE**	5.556	-5.873 to 16.99	No	ns	0.7172
**CTRL+A2A AGONIST VS. AC-HBO$_2$ +A2A AGONIST**	8.020	-6.202 to 22.24	No	ns	0.5729
**CTRL+A2A AGONIST VS. IN-HBO$_2$ BASELINE**	-4.274	-15.65 to 7.104	No	ns	0.8825
**CTRL+A2A AGONIST VS. IN-HBO$_2$ +A2A AGONIST**	-4.416	-18.64 to 9.806	No	ns	0.9444

**AC-HBO$_2$ BASELINE VS. AHBO+A2A AGONIST**	2.464	-8.966 to 13.89	No	ns	0.9887
**AC-HBO$_2$ BASELINE VS. IN-HBO$_2$ BASELINE**	-9.830	-17.43 to -2.227	Yes	**	0.0039
**AC-HBO$_2$ BASELINE VS. IN-HBO$_2$ +A2A AGONIST**	-9.972	-21.40 to 1.457	No	ns	0.1233
**AC-HBO$_2$ +A2A AGONIST VS. IN-HBO$_2$ BASELINE**	-12.29	-23.67 to -0.9165	No	ns	0.1218
**AC-HBO$_2$ +A2A AGONIST VS. IN-HBO$_2$ +A2A AGONIST**	-12.44	-26.66 to 1.786	Yes	*	0.0264
**IN-HBO$_2$ BASELINE VS. IN-HBO$_2$ +A2A AGONIST**	-0.1419	-11.52 to 11.24	No	ns	>0.9999

Figure 5d

Table Analyzed	A1 antagonist through groups (hypoxia)
Data sets analyzed	A-F
ANOVA summary	
F	4.716
P value	0.0007
P value summary	***
Significant diff. among means (P < 0.05)?	Yes
R square	0.2113

TUKEY'S MULTIPLE COMPARISONS TEST	MEAN DIFF.	95.00% CI OF DIFF.	SIGNIFICANT?	SUMMARY	ADJUSTED P VALUE
**CTRL BASELINE VS. CTRL + A1 ANTAGONIST**	8.123	-4.669 to 20.91	No	ns	0.4397
**CTRL BASELINE VS. AC-HBO$_2$ BASELINE**	6.602	-1.439 to 14.64	Yes	*	0.0330
**CTRL BASELINE VS. AC-HBO$_2$ + A1 ANTAGONIST**	6.929	-5.104 to 18.96	No	ns	0.5501

**CTRL BASELINE VS. IN-HBO$_2$ BASELINE**	-3.228	-11.19 to 4.731	No	ns	0.8444
**CTRL BASELINE VS. IN-HBO$_2$ + A1 ANTAGONIST**	-6.887	-18.92 to 5.146	No	ns	0.5567
**CTRL + A1 ANTAGONIST VS. AC-HBO$_2$ BASELINE**	-1.520	-14.36 to 11.32	No	ns	0.9993
**CTRL + A1 ANTAGONIST VS. AC-HBO$_2$ + A1 ANTAGONIST**	-1.194	-16.85 to 14.46	No	ns	>0.9999
**CTRL + A1 ANTAGONIST VS. IN-HBO$_2$ BASELINE**	-11.35	-24.14 to 1.441	No	ns	0.1118
**CTRL + A1 ANTAGONIST VS. IN-HBO$_2$ + A1 ANTAGONIST**	-15.01	-30.66 to 0.6454	No	ns	0.0680
**AC-HBO$_2$ BASELINE VS. AC-HBO$_2$ + A1 ANTAGONIST**	0.3264	-11.76 to 12.41	No	ns	>0.9999
**AC-HBO$_2$ BASELINE VS. IN-HBO$_2$ BASELINE**	-9.830	-17.87 to -1.789	Yes	**	0.0076
**AC-HBO$_2$ BASELINE VS. IN-HBO$_2$ + A1 ANTAGONIST**	-13.49	-25.58 to -1.402	No	ns	0.0596
**AC-HBO$_2$ + A1 ANTAGONIST VS. IN-HBO$_2$ BASELINE**	-10.16	-22.19 to 1.876	No	ns	0.1478
**AC-HBO$_2$ A1 ANTAGONIST VS. IN-HBO$_2$ + A1 ANTAGONIST**	-13.82	-28.86 to 1.225	No	ns	0.0905
**IN-HBO$_2$ BASELINE VS. IN-HBO$_2$ + A1 ANTAGONIST**	-3.659	-15.69 to 8.374	No	ns	0.9489

Figure 5e

Table Analyzed	A2a antagonist through groups (hypoxia)
Data sets analyzed	A-F
ANOVA summary	
F	3.508
P value	0.0061

P value summary	**
Significant diff. among means ($P < 0.05$)?	Yes
R square	0.1647

TUKEY'S MULTIPLE COMPARISONS TEST	MEAN DIFF.	95.00% CI OF DIFF.	SIGNIFICANT?	SUMMARY	ADJUSTED P VALUE
**CTRL BASELINE VS. CTRL + A2A ANTAGONIST**	7.300	-4.204 to 18.80	No	ns	0.4407
**CTRL BASELINE VS. AC-HBO_2 BASELINE**	6.602	-1.086 to 14.29	Yes	*	0.0330
**CTRL BASELINE VS. AC-HBO_2 + A2A ANTAGONIST**	3.430	-8.074 to 14.93	No	ns	0.9530
**CTRL BASELINE VS. IN-HBO_2 BASELINE**	-3.228	-10.84 to 4.381	No	ns	0.8182
**CTRL BASELINE VS. IN-HBO_2 + A2A ANTAGONIST**	1.807	-9.697 to 13.31	No	ns	0.9974
**CTRL A2A ANTAGONIST VS. AC-HBO_2 BASELINE**	-0.6978	-12.25 to 10.86	No	ns	>0.9999
**CTRL A2A ANTAGONIST VS. AC-HBO_2 + A2A ANTAGONIST**	-3.870	-18.25 to 10.51	No	ns	0.9696
**CTRL A2A ANTAGONIST VS. IN-HBO_2 BASELINE**	-10.53	-22.03 to 0.9762	No	ns	0.0926
**CTRL A2A ANTAGONIST VS. IN-HBO_2 + A2A ANTAGONIST**	-5.493	-19.87 to 8.888	No	ns	0.8750
**AC-HBO_2 BASELINE VS. AC-HBO_2 + A2A ANTAGONIST**	-3.172	-14.73 to 8.384	No	ns	0.9669
**AC-HBO_2 BASELINE VS. IN-HBO_2 BASELINE**	-9.830	-17.52 to -2.142	Yes	**	0.0045
**AC-HBO_2 BASELINE VS. IN-HBO_2 + A2A ANTAGONIST**	-4.795	-16.35 to 6.762	No	ns	0.8316
**AC-HBO_2 + A2A ANTAGONIST VS. IN-HBO_2 BASELINE**	-6.658	-18.16 to 4.846	No	ns	0.5448

**AC-HBO$_2$ + A2A ANTAGONIST VS. IN-HBO$_2$ + A2A ANTAGONIST**	-1.623	-16.00 to 12.76	No	ns	0.9995
**IN-HBO$_2$ BASELINE VS. IN-HBO$_2$ + A2A ANTAGONIST**	5.035	-6.469 to 16.54	No	ns	0.7978

Figure 6. The relative gene expression of *A1R* (a) and *A2aR* (b) genes in cerebral blood vessels from the surface of the brain of CTRL, Ac-HBO_2 and In-HBO_2 groups of rats determined by RTqPCR method. Results are presented as mean relative mRNA expressions normalized to the expression of HPRT1 housekeeping gene. Significant differences were assessed as *p < 0.05, One-Way ANOVA test was performed.

Figure 6a

Table Analyzed	Data 1
Data sets analyzed	A-C
ANOVA summary	
F	4.908
P value	0.0243
P value summary	*
Significant diff. among means (P < 0.05)?	Yes
R square	0.4121

TUKEY'S MULTIPLE COMPARISONS TEST	MEAN DIFF.	95.00% CI OF DIFF.	SIGNIFICANT?	SUMMARY	ADJUSTED P VALUE
**A1 CTRL VS. A1 AC-HBO_2**	-0.06845	-0.1544 to 0.01755	No	ns	0.1294
**A1 CTRL VS. A1 IN-HBO_2**	-0.1051	-0.1953 to -0.01491	Yes	*	0.0221
**A1 AC-HBO VS. A1 IN-HBO_2**	-0.03666	-0.1269 to 0.05353	No	ns	0.5508

Figure 6b

Table Analyzed	Data 2
Data sets analyzed	A-C
ANOVA summary	
F	11.44
P value	0.0010
P value summary	***

Significant diff. among means ($P < 0.05$)?	Yes
R square	0.6039

TUKEY'S MULTIPLE COMPARISONS TEST	MEAN DIFF.	95.00% CI OF DIFF.	SIGNIFICANT?	SUMMARY	ADJUSTED P VALUE
**A2 CTRL VS. A2 AC-HBO_2**	-0.01095	-0.08919 to 0.06729	No	ns	0.9301
**A2 CTRL VS. A2 IN-HBO_2**	-0.1299	-0.2081 to -0.05163	Yes	**	0.0017
**A2 AC-HBO_2 VS. A2 IN-HBO_2**	-0.1189	-0.1972 to -0.04068	Yes	**	0.0035

Figure 7. Relative protein expression and representative blots of A1R (A) and A2aR (B) in surface cerebral blood vessels of CTRL, Ac-HBO_2 and In-HBO_2 groups of rats determined by Western blot method. Images were taken on Bio-Rad ChemiDoc imager and analyzed by the ImageJ program. Results are presented as mean relative protein expressions normalized to the expression of β-actin. Significant differences were assessed as $^{*\#}p<0.05$, Ac-HBO_2 compared to CTRL and In-HBO_2, respectively, One-WayANOVA test was performed. Data are presented as means ± SD.

Figure 7a

	HBO_adenosine
Table Analyzed	A1R
Data sets analyzed	A-C
ANOVA summary	
F	5.051
P value	0.0210
P value summary	*
Significant diff. among means (P < 0.05)?	Yes
R square	0.4024

HOLM-SIDAK'S MULTIPLE COMPARISONS TEST	MEAN DIFF.	SIGNIFICANT?	SUMMARY	ADJUSTED P VALUE
**AC-HBO_2 VS. CTRL**	-0.1442	No	ns	0.2059
**IN-HBO_2 VS. CTRL**	0.2009	No	ns	0.1634
**IN-HBO_2 VS. AC-HBO_2**	0.3451	Yes	*	0.0191

Figure 7b

	HBO_adenosine
Table Analyzed	A2aR
Data sets analyzed	A-C

ANOVA summary	
F	49.80
P value	<0.0001
P value summary	****
Significant diff. among means (P < 0.05)?	Yes
R square	0.8691

HOLM-SIDAK'S MULTIPLE COMPARISONS TEST	MEAN DIFF.	SIGNIFICANT?	SUMMARY	ADJUSTED P VALUE
**AC-HBO_2 VS. CTRL**	-0.4453	Yes	****	<0.0001
**IN-HBO_2 VS. CTRL**	0.03705	No	ns	0.5016
**IN-HBO_2 VS. AC-HBO_2**	0.4824	Yes	****	<0.0001
